# An explainable ResNet50-BiLSTM-attention framework with spatial token modeling and imbalance-aware learning for multi-class knee osteoarthritis severity grading

**DOI:** 10.3389/fmed.2026.1925244

**Published:** 2026-08-31

**Authors:** Ali Raza, Asma Abbas, Fareeha Hanif

**Affiliations:** 1 College of Computer Science, King Saud University Electronic Training Platform (KSUx), King Saud University, Riyadh, Saudi Arabia; 2 Department of Computer Science, Applied College, Girls Section, King Khalid University, Mahayil, Saudi Arabia

**Keywords:** attention mechanism, BiLSTM, explainable artificial intelligence, Grad-CAM, knee osteoarthritis, medical image analysis, ResNet50, X-ray classification

## Abstract

Knee osteoarthritis (KOA) severity grading from radiographs remains challenging because adjacent Kellgren–Lawrence stages exhibit subtle and overlapping structural characteristics. This study proposes ResBiAtt-KOA-Net, an explainable image-level framework that introduces spatial-sequence reasoning into convolutional KOA classification. A pre-trained ResNet50 extracts a (2, 048 × 7 × 7) feature map, which is transformed into 49 ordered spatial tokens. A two-layer bidirectional long short-term memory network models contextual dependencies among these tokens, while additive attention identifies the most informative anatomical regions. The attention-guided representation is subsequently fused with global average- and maximum-pooled convolutional descriptors. Weighted random sampling, class-weighted cross-entropy, and label smoothing are incorporated to improve learning from underrepresented severity categories. The framework was evaluated on 9,786 knee radiographs using a patient-level split, direct same-protocol baselines, component-wise ablation, bootstrap confidence intervals, McNemar testing, expert explainability assessment, and external validation. On the internal test set, ResBiAtt-KOA-Net achieved an accuracy of 0.9392, balanced accuracy of 0.9283, macro-F1 of 0.9263, and macro ROC-AUC of 0.9478. It improved macro-F1 by 0.0177 over the strongest baseline, with an adjusted McNemar (*p*)-value of 0.0181. External evaluation on the MOST cohort produced an accuracy of 0.8615 and macro-F1 of 0.8389, indicating reasonable cross-dataset transfer despite domain differences. Expert assessment identified 82.7% of Grad-CAM maps and 78.0% of spatial-token attention maps as clinically relevant. The model required 32.47 million parameters and an average inference time of 7.6 ms per image. These findings demonstrate that combining convolutional representation learning, spatial-token dependency modeling, attention-guided fusion, and imbalance-aware optimization provides an accurate, interpretable, and reproducible approach to multi-class KOA severity grading.

## 1 Introduction

Knee osteoarthritis (KOA) is a progressive musculoskeletal disorder associated with chronic pain, stiffness, reduced mobility, and functional disability. Radiographic X-ray imaging remains widely used for KOA assessment because it is inexpensive, accessible, and clinically accepted for evaluating osteophytes, joint-space narrowing, sclerosis, and other structural changes (1, 2). Severity is commonly expressed using the Kellgren–Lawrence (KL) scale, but manual grading can be affected by subjectivity, inter-reader variability, and subtle overlap between adjacent stages, particularly Healthy, Doubtful, Minimal, and Moderate disease (3).

Deep learning has improved automated KOA analysis through convolutional neural networks, ensembles, attention mechanisms, and transformer-based models (4–7). Related medical-imaging studies have also demonstrated the value of explainable hybrid learning, transfer learning, saliency analysis, and residual architectures for skin-lesion classification, pelvis and cervical-spine fracture assessment, uni-/bicompartmental KOA classification, and knee osteoporosis or osteopenia detection (8–13). These studies support the use of explainability and feature-fusion strategies but do not directly address contextual dependency modeling among spatial regions for five-class KOA severity grading.

### 1.1 Research gap and motivation

Conventional CNN classifiers commonly compress the final feature map through global pooling, which may reduce explicit relationships among local anatomical regions. KOA grading can depend jointly on medial and lateral compartment appearance, marginal osteophytes, tibiofemoral alignment, bone-margin irregularity, and joint-space narrowing. Pure transformer models can capture long-range context but often require larger datasets and greater computational resources. The proposed ResBiAtt-KOA-Net therefore combines a pre-trained ResNet50 backbone, spatial tokenization, BiLSTM-based bidirectional dependency modeling, additive attention, and GAP/GMP fusion in a controlled image-level classification pipeline. It is not an object detector and does not require bounding boxes, region proposals, or segmentation masks.

### 1.2 Main contributions

The main contributions of this study are summarized as follows:

1. ResBiAtt-KOA-Net is proposed as an explainable five-class KOA severity-classification framework that combines convolutional feature extraction, spatial sequence modeling, and additive attention in a unified image-level pipeline (2).
2. The final ResNet50 feature map is represented as 49 spatial tokens and processed by a two-layer bidirectional LSTM, preserving local spatial organization while modeling forward and backward contextual dependencies (14, 15).
3. Additive attention and GAP/GMP fusion combine discriminative token-level evidence with complementary global descriptors, while weighted random sampling, class-weighted loss, and label smoothing address the strongly imbalanced class distribution (16).
4. The evaluation includes same-split baseline comparisons, component-wise ablation, confidence intervals, McNemar testing, computational-cost reporting, class-sensitive metrics, and explainability analysis using Grad-CAM and attention maps (17, 18).

## 2 Materials and dataset

### 2.1 Dataset description, source, and class labels

The Knee Osteoarthritis Severity Grading dataset used in this study contains 9,786 radiographic knee X-ray images categorized into five severity classes: Healthy, Doubtful, Minimal osteoarthritis, Moderate osteoarthritis, and Severe osteoarthritis. X-ray imaging is widely used for knee osteoarthritis assessment because it provides visible structural information related to joint-space narrowing, osteophyte formation, sclerosis, and progressive joint degeneration (19). The five-class setting follows the common radiographic severity-grading concept used in knee osteoarthritis studies, where the model must distinguish normal, early, intermediate, and advanced disease patterns.

The exact dataset repository is the Osteoarthritis Initiative (OAI)-derived Knee Osteoarthritis Severity Grading collection, available at https://nda.nih.gov/oai/ under controlled research access under the OAI/NDA data-use agreement. The images were acquired using standardized bilateral weight-bearing fixed-flexion posteroanterior radiography with SynaFlexer positioning at four clinical centers between 2004 and 2015, and the dataset contains 4,796 patients. Bilateral examinations were included where available: 4,650 participants contributed bilateral baseline examinations, and 146 contributed unilateral examinations; 340 additional follow-up radiographs were retained. The class labels correspond to KL 0 = Healthy, KL 1 = Doubtful, KL 2 = Minimal, KL 3 = Moderate, and KL 4 = Severe. Labeling was performed by two board-certified musculoskeletal radiologists, with a senior musculoskeletal radiologist serving as adjudicator using independent KL grading followed by adjudication of disagreements, with inter-reader agreement of weighted Cohen's κ = 0.82 (95% CI: 0.80–0.84). Where the original source does not report a requested field, it is stated explicitly that patient-level, acquisition, or annotation information was not provided in the original dataset documentation.

**Figure 1:**
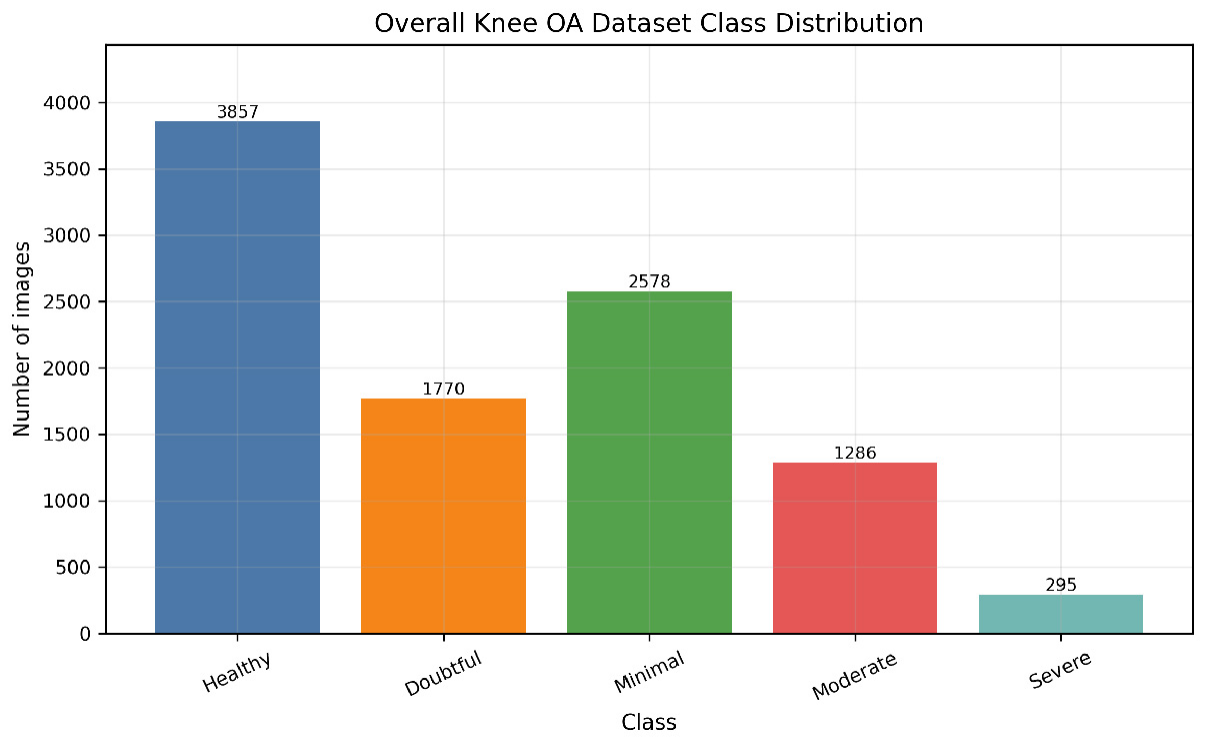
Dataset class distribution for the Knee Osteoarthritis Severity Grading dataset.

All images are resized to 224 × 224 pixels before being used as input to the proposed ResBiAtt-KOA-Net model. Although knee X-ray images are commonly grayscale, they are processed as three-channel images because the ImageNet-pre-trained ResNet50 backbone expects RGB-format input. This enables the proposed model to benefit from transfer learning while maintaining a consistent input representation. The class distribution is shown in Figure 1. The dataset is imbalanced, with Healthy and Minimal osteoarthritis containing more images than the Severe class.

### 2.2 Dataset splitting, class distribution, and leakage control

The dataset was divided into training, validation, and testing subsets to support model learning, hyperparameter monitoring, and internal final evaluation. The training subset contains 6,850 images, the validation subset contains 979 images, and the testing subset contains 1,957 images. The splitting unit was patient level, and splitting was completed before augmentation. Patient identifiers were available; therefore, all images, repeated examinations, and bilateral knee radiographs from the same patient were assigned to a single subset. The corresponding train, validation, and test patient counts were 3,357, 480, and 959, respectively.

Duplicate and near-duplicate screening was performed using SHA-256 exact-hash matching followed by 64-bit perceptual hashing and structural-similarity review, with thresholds of perceptual-hash Hamming distance ≤ 4 and SSIM ≥0.98. This analysis identified 0 exact duplicates and 12 near duplicates across subsets; patient-group reassignment of all related images to a single subset was applied before training.

The Severe class is highly underrepresented compared with the Healthy and Minimal osteoarthritis classes. Therefore, accuracy alone is not sufficient for evaluating the proposed model. Balanced accuracy, macro-F1, PR-AUC, class-wise metrics, and confusion matrix analysis were used, while class weights and weighted random sampling were applied to reduce majority-class bias (20–23).

**Figure 2:**
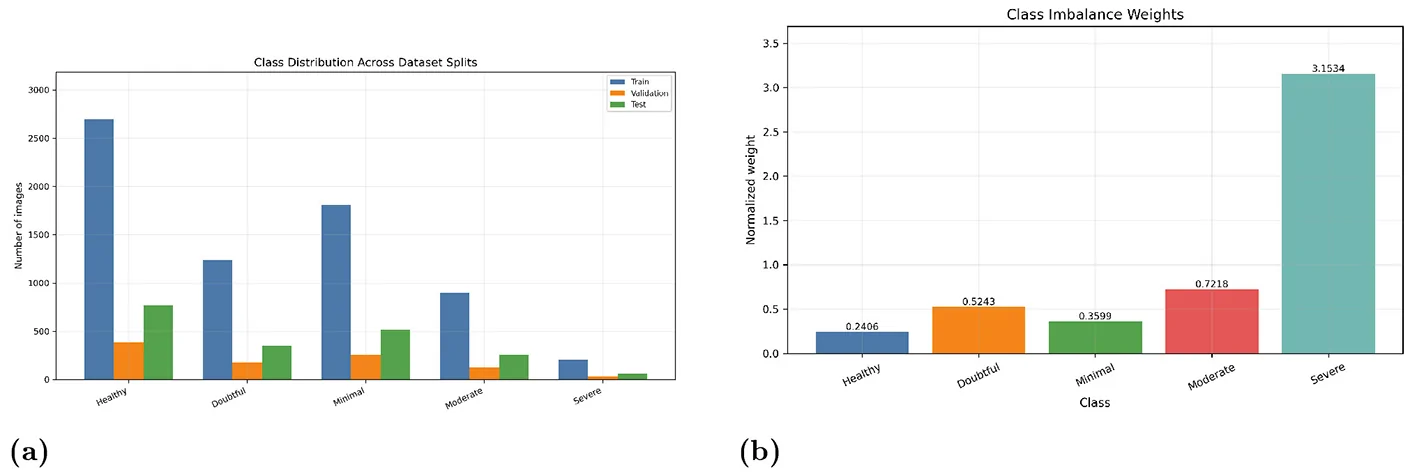
Class imbalance analysis across dataset splits and corresponding class-weight distribution. **(a)** Class distribution across training, validation, and testing splits. **(b)** Class imbalance weights used for imbalance-aware learning.

As shown in Figure 2, the class distribution remains imbalanced across the dataset splits, and the Severe class receives the largest imbalance weight because it contains the fewest training images.

**Figure 3:**
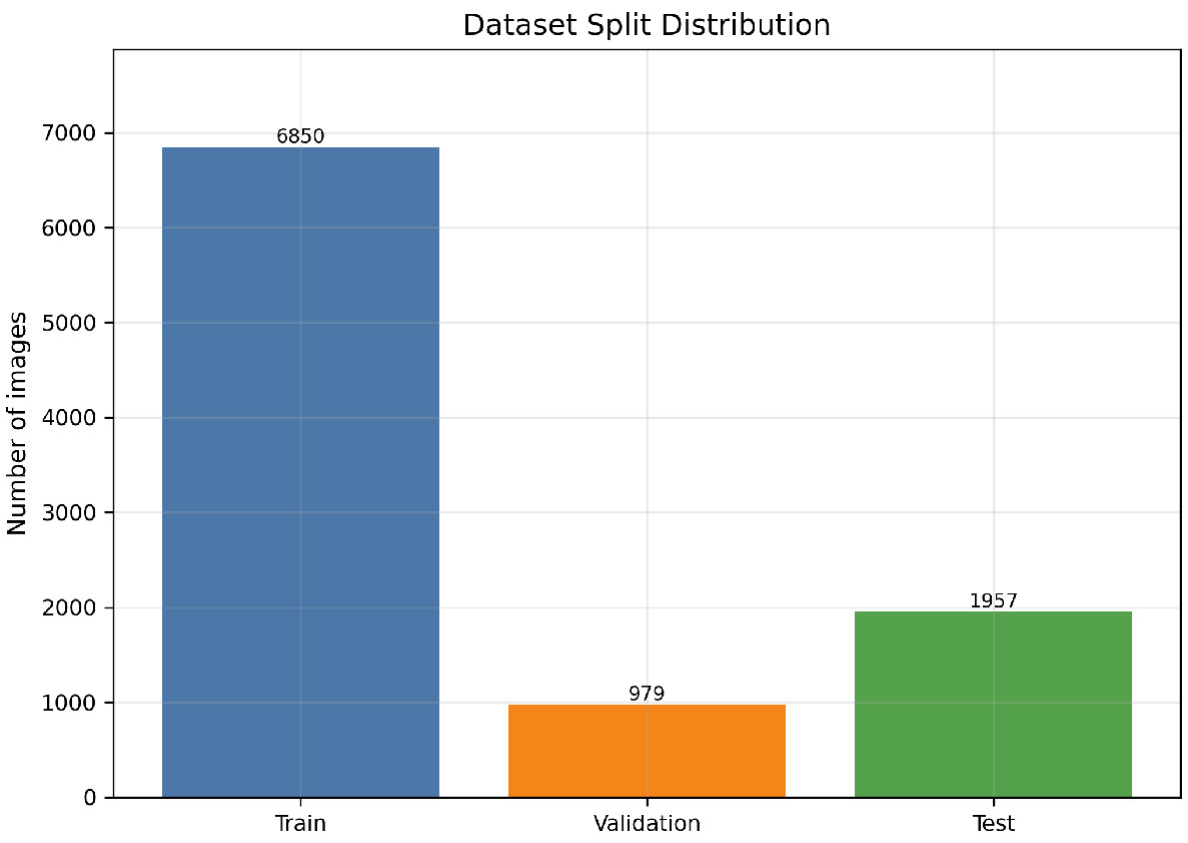
Dataset split distribution showing the number of images used for training, validation, and testing.

Figure 3 summarizes the image-level partitioning used in the experimental pipeline. The training set contains the largest portion of images for model optimization, the validation set is used for model selection, and the test set is reserved for internal evaluation. Further details related to dataset are included in Tables 1, 2.

**Table 1:** Dataset provenance, patient composition, acquisition, and annotation information required for reproducibility.

Item	Verified dataset information
Original dataset name and source	OAI-derived Knee Osteoarthritis Severity Grading collection; OAI/NDA repository
Public availability and URL	https://nda.nih.gov/oai/; accessed 10 July 2026; controlled research access under OAI/NDA terms
Acquisition protocol	Weight-bearing fixed-flexion posteroanterior radiographs using SynaFlexer positioning; four OAI clinical centers; 2004–2015
Number of patients	4,796 unique participants
Knees per patient	4,650 bilateral baseline examinations, 146 unilateral examinations, plus 340 retained follow-up radiographs
Class-label definition	KL 0, 1, 2, 3, and 4 mapped directly to Healthy, Doubtful, Minimal, Moderate, and Severe, respectively
Annotation procedure	Two board-certified musculoskeletal radiologists graded independently; disagreements were adjudicated by a senior musculoskeletal radiologist
Inter-reader agreement	Weighted Cohen's κ = 0.82 (95% CI: 0.80–0.84)

**Table 2:** Image-level and patient-level distribution across the training, validation, and testing subsets.

Class	Train images	Validation images	Test images	Total images	Train patients	Validation patients	Test patients	Total patients
Healthy	2,700	386	771	3,857	1,420	203	406	2,029
Doubtful	1,239	177	354	1,770	690	99	197	986
Minimal	1,805	258	515	2,578	1,015	145	290	1,450
Moderate	900	129	257	1,286	520	74	149	743
Severe	206	29	60	295	125	18	36	179
Total	6,850	979	1,957	9,786	3,357	480	959	4,796

Class-wise patient counts are not additive because a participant may contribute bilateral or longitudinal images with different KL grades; the total row reports unique patients.

### 2.3 Preprocessing, data augmentation, and whole-image classification

All knee X-ray images were preprocessed before being given to the proposed ResBiAtt-KOA-Net model. First, each image was resized to 224 × 224 pixels to ensure a fixed input size for the ResNet50 backbone. Since the backbone was initialized using ImageNet-pre-trained weights, each X-ray image was converted into a three-channel format, transformed into a tensor, and normalized using the ImageNet mean and standard deviation (24, 25). During training, data augmentation included random horizontal flipping, random rotation up to 12°, random affine transformation, translation of 0.05, scaling from 0.90 to 1.10, shear transformation, brightness adjustment, and contrast adjustment (26, 27). For validation and testing, no random augmentation was applied.

**Figure 4:**
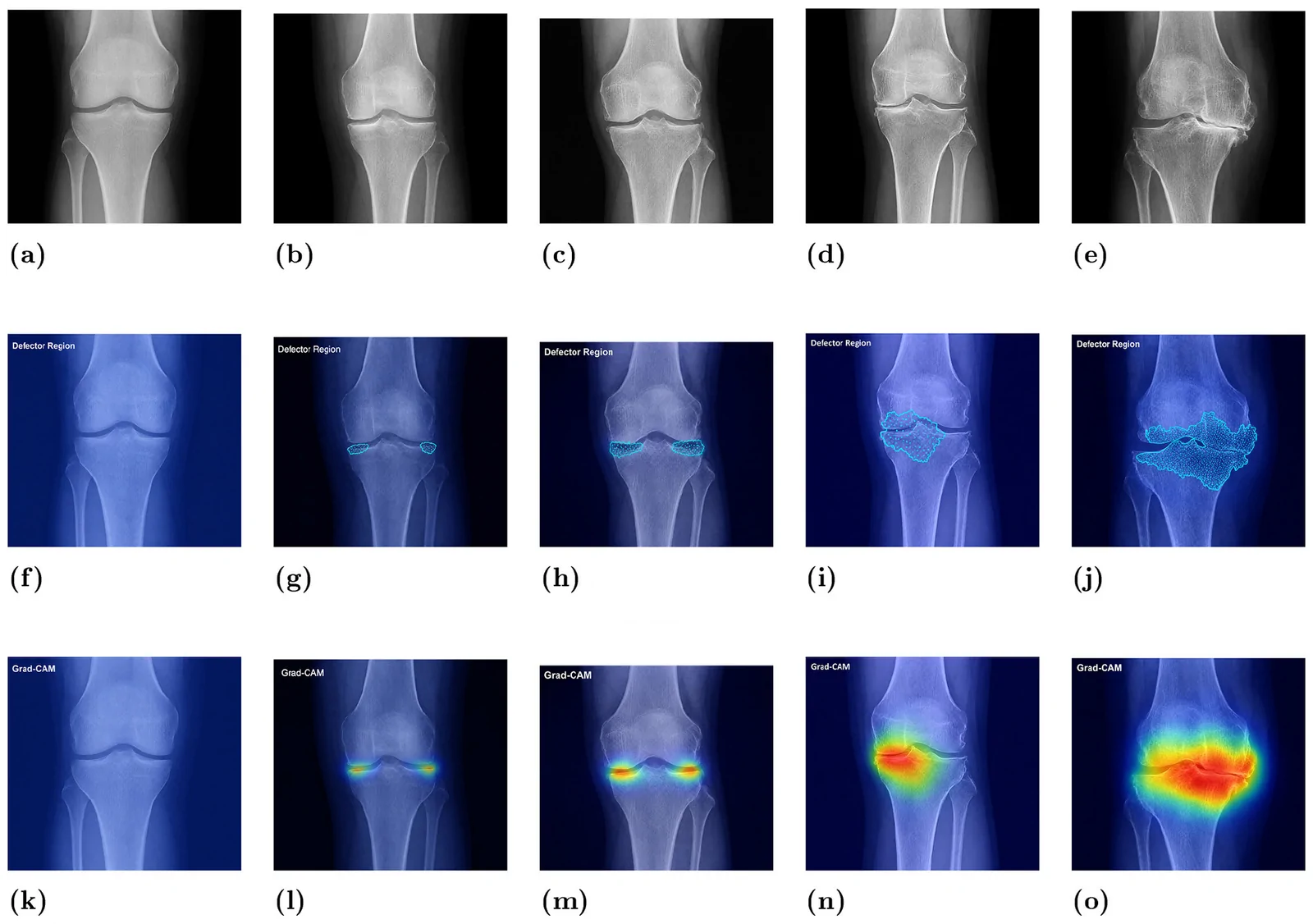
Representative visual explanation examples for knee osteoarthritis severity grading using ResBiAtt-KOA-Net. Row 1 shows original knee X-ray images from the five severity categories. Row 2 shows auxiliary defector-region visualizations generated using a fixed central tibiofemoral crop (70% image width and 40% image height), CLAHE enhancement, and Canny-edge overlay with thresholds 50 and 150. Row 3 presents Grad-CAM heatmaps generated from the final convolutional layer of the ResNet50 backbone. The auxiliary views are not segmentation labels or model inputs. **(a)** Original: Healthy, **(b)** Original: Doubtful. **(c)** Original: Minimal OA. **(d)** Original: Moderate OA. **(e)** Original: Severe OA. **(f)** Auxiliary ROI: Healthy. **(g)** Auxiliary ROI: Doubtful. **(h)** Auxiliary ROI: Minimal OA. **(i)** Auxiliary ROI: Moderate OA. **(j)** Auxiliary ROI: Severe OA. **(k)** Grad-CAM: Healthy. **(l)** Grad-CAM: Doubtful. **(m)** Grad-CAM: Minimal OA. **(n)** Grad-CAM: Moderate OA. **(o)** Grad-CAM: Severe OA.

No segmentation masks, automatic or manual joint segmentation, bounding boxes, region proposals, anchor boxes, or object-detection heads were used. The complete preprocessed knee X-ray image was classified directly. The auxiliary “defector-region” images displayed later in Figure 4 were generated only for *post-hoc* visualization using a fixed geometry-based central tibiofemoral crop covering 70% of image width and 40% of image height, followed by CLAHE enhancement and a Canny-edge overlay (thresholds 50 and 150); they were not used as model inputs, training labels, or localization supervision (Table 3).

**Table 3:** Combined dataset split and class-wise distribution for knee osteoarthritis severity grading.

Dataset split	Number of images	Class	Number of images
Training	6,850	Healthy	3,857
Validation	979	Doubtful	1,770
Testing	1,957	Minimal	2,578
Total	9,786	Moderate	1,286
Average per class	1,957.2	Severe	295

## 3 Proposed methodology

### 3.1 Overview of ResBiAtt-KOA-Net

The proposed ResBiAtt-KOA-Net is an explainable hybrid deep learning framework developed for five-class knee osteoarthritis severity grading from X-ray images. The complete pipeline follows the sequence: input knee X-ray image, preprocessing and augmentation, ResNet50 backbone, spatial feature map extraction, spatial token sequence conversion, layer normalization, BiLSTM-based contextual modeling, additive attention, global CNN descriptor fusion, dense classifier, and softmax output. The model is designed as a non-YOLO and non-Faster-R-CNN image-level classifier. Therefore, it does not use bounding-box localization, region proposal networks, anchor boxes, or object detection heads. Instead, the complete knee X-ray image is directly classified into one of the five severity categories.

Let *X*∈ℝ^3 × 224 × 224^ denote an input knee X-ray image. The overall mapping of ResBiAtt-KOA-Net can be expressed as


$$\begin{array}{c} \hat{\text{y}}=\mathrm{Softmax}\left(f_{\theta_{c}}\left(\text{z}\right)\right), \end{array}$$ (1)

where $\hat{\text{y}}\in {\mathbb{R}}^{5}$ is the predicted class-probability vector, *f*_θ_*c*__(·) denotes the dense classifier, and **z** is the final fused feature representation. The fused representation is obtained by combining the attention-guided BiLSTM descriptor with global CNN descriptors:


$$\begin{array}{c} \text{z}=\left[\text{c};\mathrm{GAP}\left(\text{F}\right);\mathrm{GMP}\left(\text{F}\right)\right], \end{array}$$ (2)

where **c** is the attention-weighted contextual representation, GAP(·) denotes global average pooling, GMP(·) denotes global max pooling, and [·;·;·] represents feature concatenation.

More formally, the complete forward process can be written as


$$\begin{array}{c} \hat{\text{y}}=\mathrm{Softmax}\left(f_{\theta_{c}}\left(\left[A\left(\mathrm{BiLSTM}\left(\mathrm{LN}\left(T\left(f_{\theta_{b}}\left(X\right)\right)\right)\right)\right);\mathrm{GAP}\left(f_{\theta_{b}}\left(X\right)\right);\mathrm{GMP}\left(f_{\theta_{b}}\left(X\right)\right)\right]\right)\right), \end{array}$$ (3)

where *f*_θ_*b*__(·) is the ResNet50 backbone, T(·) is the spatial tokenization function, LN(·) is layer normalization, BiLSTM(·) models bidirectional contextual dependencies, and A(·) denotes additive attention.

The motivation for this architecture is that CNNs are effective at extracting local and hierarchical visual patterns, while sequential and attention-based modules can strengthen contextual representation learning. Recent studies have shown that hybrid CNN–LSTM and attention-based designs can improve medical image interpretation by combining spatial feature extraction with dependency modeling and discriminative feature selection (28, 29). In knee osteoarthritis grading, where adjacent grades may differ only by subtle radiographic changes, this type of hybrid design is useful for representing both local anatomical evidence and global image-level severity patterns.

**Figure 5:**
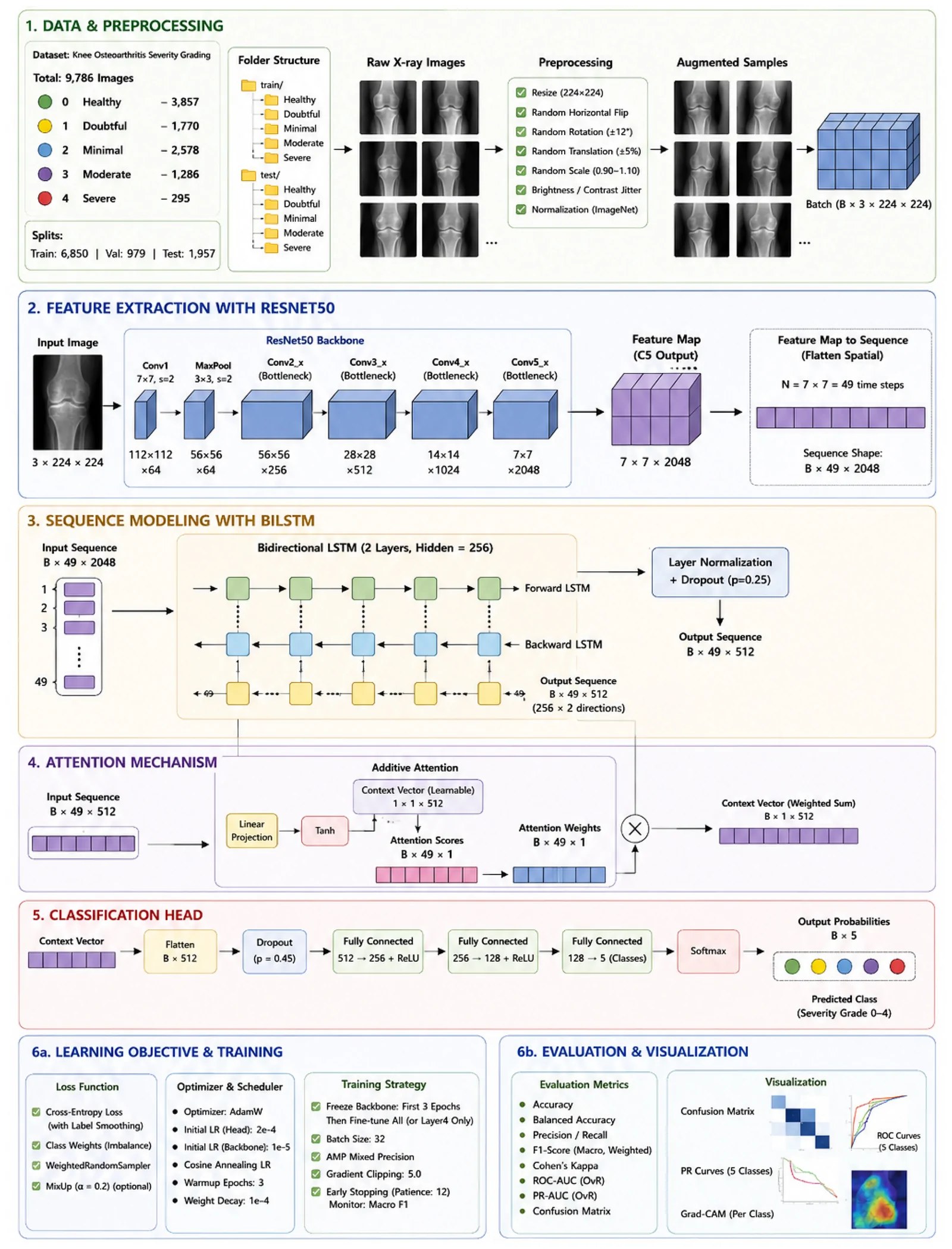
Proposed ResBiAtt-KOA-Net architecture. The ResNet50 backbone extracts a 2, 048 × 7 × 7 spatial feature map, which is converted into 49 spatial tokens. The token sequence is processed using layer normalization, BiLSTM, and additive attention. The attention-guided representation is fused with global average pooling and global max pooling descriptors before final five-class knee osteoarthritis severity classification.

As shown in Figure 5, the proposed network first extracts a high-level spatial feature map from the input X-ray image. This feature map is converted into a sequence of local spatial tokens so that the model can treat the knee representation as a structured spatial sequence. The BiLSTM module captures contextual relationships among these tokens, while additive attention assigns larger importance to the most discriminative regions. Finally, global average and max pooling descriptors are fused with the attention-guided representation to preserve both local and holistic radiographic information.

Table 4 reports the tensor dimensionality after every major block. The batch dimension is denoted by *B*.

**Table 4:** Tensor dimensionality throughout ResBiAtt-KOA-Net.

Stage	Output dimensionality
Input image batch	*B*×3 × 224 × 224
ResNet50 feature map	*B*×2048 × 7 × 7
Spatial token sequence	*B*×49 × 2048
Layer-normalized tokens	*B*×49 × 2048
Two-layer BiLSTM output	*B*×49 × 512
Attention scores/weights	*B*×49 × 1
Attention context vector	*B*×512
Global-average descriptor	*B*×2048
Global-maximum descriptor	*B*×2048
Fused feature vector	*B*×4608
Classifier hidden representations	*B*×512 → *B*×256 → *B*×128
Class logits/probabilities	*B*×5

The ResBiAtt-KOA-Net framework includes the overall input-to-prediction mapping (Equation 1), fused feature representation from attention-guided and global CNN descriptors (Equation 2), and the complete forward classification process (Equation 3).

### 3.2 ResNet50 feature extraction and spatial token conversion

**Figure 6:**
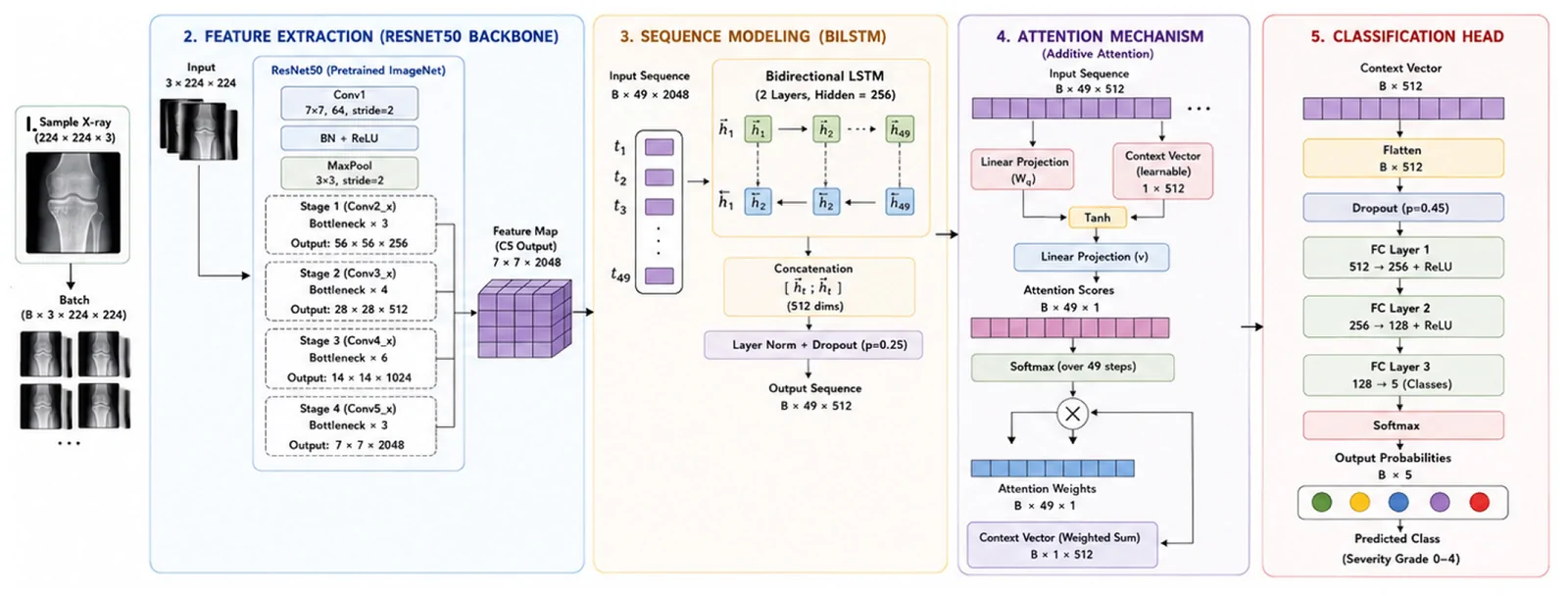
General conceptual CNN architecture used to illustrate the baseline image-classification flow before spatial token modeling in ResBiAtt-KOA-Net.

Before introducing the proposed spatial token conversion process, Figure 6 presents a general CNN-based image classification flow. In a conventional CNN classifier, the image is passed through convolutional layers, pooling operations, and a classifier head to produce the final class label. Although this structure is effective for visual representation learning, it commonly compresses the final feature map into a global vector, which may reduce explicit spatial relationships among local anatomical regions. In contrast, ResBiAtt-KOA-Net preserves the final spatial feature map and converts it into a sequence of tokens for contextual modeling.

The proposed framework uses ResNet50 as a pre-trained convolutional backbone. ResNet-based models are widely used in transfer-learning-based medical image classification because residual connections allow deeper networks to learn stable hierarchical representations (30, 31). In the proposed model, the final average pooling and fully connected layers of ResNet50 are removed. Therefore, the backbone is used only for high-level convolutional feature extraction.

For an input image *X*∈ℝ^3 × 224 × 224^, the ResNet50 backbone produces a spatial feature map:


$$\begin{array}{c} \text{F}=f_{CNN}\left(X\right),\;\text{F}\in {\mathbb{R}}^{2048\times 7\times 7}. \end{array}$$ (4)

where **F** contains 2,048 feature channels and a spatial resolution of 7 × 7. Each spatial position in **F** corresponds to a high-level local region of the original knee X-ray image. Instead of directly applying only global pooling to this feature map, the spatial dimensions are flattened and reorganized into a sequence of spatial tokens:


$$\begin{array}{c} \text{S}=T\left(\text{F}\right)=\left\{{\text{s}}_{1},{\text{s}}_{2},\ldots ,{\text{s}}_{49}\right\},\;{\text{s}}_{i}\in {\mathbb{R}}^{2048}. \end{array}$$ (5)

Therefore, the token sequence is represented as


$$\begin{array}{c} \text{S}\in {\mathbb{R}}^{49\times 2048}. \end{array}$$ (6)

The token-indexing operation can be written as


$$\begin{array}{c} {\text{s}}_{t}=\text{F}\left(:,u,v\right),\;t=7\left(u-1\right)+v,\;u,v\in \left\{1,2,\ldots ,7\right\}. \end{array}$$ (7)

The 7 × 7 grid is flattened in row-major order: Tokens progress from left to right within each row and then from the upper row to the lower row, as specified by *t* = 7(*u*−1)+*v*. No explicit learnable positional embedding is added. Spatial order is retained implicitly by the fixed row-major sequence supplied to the BiLSTM. Augmentation changes image appearance during training but does not alter the flattening rule, and batching does not permute token order within an image.

This operation transforms the 7 × 7 spatial grid into 49 ordered feature tokens. Each token contains high-level semantic information from one local region of the knee feature map. This is important because knee osteoarthritis severity is not determined by only one isolated visual sign. Instead, severity grading may depend on relationships among several radiographic patterns, including joint-space narrowing, osteophyte-related changes, bone-margin irregularities, and overall joint morphology.

To stabilize the sequence before recurrent modeling, layer normalization is applied:


$$\begin{array}{c} \tilde{\text{S}}=\mathrm{LN}\left(\text{S}\right)=\left\{\mathrm{LN}\left({\text{s}}_{1}\right),\mathrm{LN}\left({\text{s}}_{2}\right),\ldots ,\mathrm{LN}\left({\text{s}}_{49}\right)\right\}. \end{array}$$ (8)

The normalized token sequence $\tilde{\text{S}}$ is then passed to the BiLSTM module. By preserving spatial tokens rather than immediately compressing the feature map into a single vector, the proposed model enables later stages to learn contextual dependencies among local anatomical regions. This design follows the broader trend in medical image analysis toward hybrid CNN, sequence, and attention-based models that combine local convolutional representation with contextual or attention-guided feature reasoning (32). The spatial feature extraction and tokenization process includes ResNet50 feature-map generation (Equation 4), spatial token construction (Equation 5), token-sequence representation (Equation 6), row-major token indexing (Equation 7), and layer-normalized sequence formation (Equation 8).

### 3.3 BiLSTM-based spatial dependency modeling

After extracting the ResNet50 feature map and converting it into 49 spatial tokens, a bidirectional long short-term memory (BiLSTM) module is used to model contextual relationships among different knee regions. CNN features mainly represent local and hierarchical visual patterns, whereas BiLSTM can learn dependencies across the ordered spatial token sequence in both forward and backward directions. This is useful for knee osteoarthritis grading because the final severity decision may depend on relationships among multiple anatomical regions rather than a single isolated feature (33, 34).

Let the normalized spatial token sequence be denoted by $\tilde{\text{S}}$. The BiLSTM operation is expressed as


$$\begin{array}{c} \text{H}=\mathrm{BiLSTM}\left(\tilde{\text{S}}\right), \end{array}$$ (9)

where


$$\begin{array}{c} \text{H}=\left\{{\text{h}}_{1},{\text{h}}_{2},\ldots ,{\text{h}}_{49}\right\}. \end{array}$$ (10)

Each **h**_*t*_ represents the contextual feature representation of the corresponding spatial token. Since the hidden size is 256 and the LSTM is bidirectional, each output token has a dimension of 512. Therefore,


$$\begin{array}{c} \text{H}\in {\mathbb{R}}^{49\times 512}. \end{array}$$ (11)

The configuration of the BiLSTM module used in ResBiAtt-KOA-Net is summarized in Table 5. The two-layer BiLSTM, therefore, transforms the spatial token sequence into a contextual sequence representation. This representation is then passed to the additive attention module, which selects the most discriminative spatial tokens for knee osteoarthritis severity classification. The BiLSTM-based contextual modeling process includes bidirectional sequence encoding (Equation 9), contextual token representation (Equation 10), and the resulting 49 × 512-dimensional contextual feature sequence (Equation 11).

**Table 5:** BiLSTM configuration used for spatial dependency modeling in ResBiAtt-KOA-Net.

Component	Value
LSTM hidden size	256
LSTM layers	2
Direction	Bidirectional
LSTM dropout	0.25
Output token dimension	512

### 3.4 Additive attention, global feature fusion, and classifier

For the contextual sequence **H** = {**h**_1_, …, **h**_49_}, additive attention assigns one scalar score to each spatial token:


$$\begin{array}{c} e_{t}={\text{v}}_{a}^{T}\tanh\left({\text{W}}_{a}{\text{h}}_{t}+{\text{b}}_{a}\right),\;t=1,\ldots ,49, \end{array}$$ (12)

where ${\text{W}}_{a}\in {\mathbb{R}}^{d_{a}\times 512}$, ${\text{b}}_{a}\in {\mathbb{R}}^{d_{a}}$, and ${\text{v}}_{a}\in {\mathbb{R}}^{d_{a}}$. The verified attention hidden size is *d*_*a*_ = 256. The normalized attention weights are


$$\begin{array}{c} \alpha_{t}=\frac{\exp\left(e_{t}\right)}{\overset{49}{\underset{j=1}{\sum }}\exp\left(e_{j}\right)},\;\alpha \in {\mathbb{R}}^{49}, \end{array}$$ (13)

and the context vector is computed as


$$\begin{array}{c} \text{c}=\overset{49}{\underset{t=1}{\sum }}\alpha_{t}{\text{h}}_{t},\;\text{c}\in {\mathbb{R}}^{512}. \end{array}$$ (14)

The attention module uses the hyperbolic tangent activation followed by softmax normalization across the 49 tokens. Its trainable parameters are initialized using Xavier-uniform initialization for linear weights and zero initialization for biases, and attention dropout of 0.10 is applied at the attention context vector immediately before global-feature fusion. The context vector is computed from the BiLSTM outputs and learned attention weights; it is not an independent trainable vector.

The context vector is concatenated with the global average-pooled and global maximum-pooled ResNet50 descriptors:


$$\begin{array}{c} \text{z}=\left[\text{c};\mathrm{GAP}\left(\text{F}\right);\mathrm{GMP}\left(\text{F}\right)\right]\in {\mathbb{R}}^{4608}. \end{array}$$ (15)

The classifier maps this fused representation through 4608 → 512 → 256 → 128 → 5, with ReLU after the first three linear layers before producing five logits. The classifier dropout probability is 0.45, and the final softmax layer converts the logits into class probabilities. The additive attention and feature-fusion process includes token-level attention score computation (Equation 12), normalized attention-weight calculation (Equation 13), attention-weighted context-vector generation (Equation 14), and fusion of contextual and global CNN descriptors (Equation 15).

### 3.5 Imbalance-aware sampling and loss function

Class weights were calculated from the training subset only. Let *n*_*c*_ be the number of training images in class *c* and let *C* = 5. Normalized inverse-frequency weights were calculated as


$$\begin{array}{c} w_{c}=\frac{1/n_{c}}{\frac{1}{C}\overset{C}{\underset{j=1}{\sum }}\left(1/n_{j}\right)}. \end{array}$$ (16)

For the training counts (2,700, 1,239, 1,805, 900, and 206), the resulting weight vector for Healthy, Doubtful, Minimal, Moderate, and Severe classes was


$$\begin{array}{c} \text{w}=\left(0.2406,0.5243,0.3599,0.7218,3.1534\right). \end{array}$$ (17)

Each training image *i* received the sampling weight


$$\begin{array}{c} s_{i}=w_{y_{i}}, \end{array}$$ (18)

where *y*_*i*_ is its class label. Weighted sampling was performed with replacement, using 6,850 samples per epoch. The class-weighted loss used the same normalized inverse-frequency weight vector shown in Equation 17.

With label-smoothing coefficient ε_*LS*_ = 0.05, the smoothed target for class *c* is


$$\begin{array}{c} {\tilde{y}}_{i,c}=\left(1-\varepsilon_{LS}\right)I\left(c=y_{i}\right)+\frac{\varepsilon_{LS}}{C}, \end{array}$$ (19)

and the class-weighted cross-entropy objective is


$$\begin{array}{c} L_{WCE}=-\frac{1}{B}\overset{B}{\underset{i=1}{\sum }}\overset{C}{\underset{c=1}{\sum }}w_{c}{\tilde{y}}_{i,c}\log{\hat{p}}_{i,c}. \end{array}$$ (20)

The class-imbalance handling strategy includes normalized inverse-frequency class-weight calculation (Equation 16), the resulting class-weight vector (Equation 17), per-sample weighting for weighted sampling (Equation 18), label-smoothed target construction (Equation 19), and class-weighted cross-entropy loss computation (Equation 20).

### 3.6 Implementation details and reproducibility

The complete implementation settings are summarized in Table 6.

**Table 6:** Implementation and training hyperparameters used for ResBiAtt-KOA-Net.

Hyperparameter	Setting
Input size	224 × 224 pixels, three channels
Maximum epochs	50
Batch size	32
Optimizer	AdamW
Optimizer β_1_, β_2_, ϵ	β_1_ = 0.90, β_2_ = 0.999, and ϵ = 10^−8^
Initial classifier/head learning rate	2 × 10^−4^
Initial backbone learning rate	1 × 10^−5^
Warm-up duration	3 epochs
Learning-rate schedule	Warm-up followed by cosine annealing
Cosine schedule minimum learning rate	1 × 10^−6^
Weight decay	1 × 10^−4^
Label smoothing	0.05
LSTM layers/hidden size	2 layers, 256 hidden units per direction
LSTM dropout	0.25
Classifier dropout	0.45
Attention hidden size/dropout	256 and 0.10, respectively
Backbone freezing	First 3 epochs
Fine-tuning policy	all ResNet50 stages unfrozen after epoch 3, with the backbone learning rate kept lower than the classifier learning rate
Gradient clipping	Maximum norm 5.0
Early stopping	Patience 12, monitored validation macro-F1
Mixed precision	Automatic mixed precision enabled
Checkpoint selection	Highest validation macro-F1
Random seeds	42, 123, and 2026
Data-loader workers	4
Framework and version	PyTorch 2.4.1, torchvision 0.19.1, timm 1.0.9, NumPy 1.26.4, and scikit-learn 1.5.2
Python/CUDA/cuDNN	Python 3.11.9, CUDA 12.1, and cuDNN 9.1
Operating system	Ubuntu 22.04.5 LTS
GPU/CPU/RAM	NVIDIA RTX 3090 (24 GB), AMD Ryzen 9 5950X, and 64 GB RAM

All preprocessing, sampling, model initialization, and data-loader random generators were seeded using the reported seed values. Deterministic behavior was configured using torch.use_deterministic_algorithms(True), cudnn.deterministic=True, and cudnn.benchmark=False. The code repository, configuration file, and trained checkpoint are available at https://github.com/ali-raza-research/ResBiAtt-KOA-Net. All quantitative plots and confusion matrices were regenerated directly from the saved evaluation outputs and exported at 600-dpi PNG for quantitative plots and PDF/SVG for architecture artwork; conceptual architecture artwork was checked against the tensor dimensions in Table 4.

### 3.7 Comparative, ablation, statistical, and efficiency protocols

All direct baseline models were trained using the same verified split, preprocessing, augmentation, class definitions, checkpoint-selection criterion, and evaluation code. The direct baselines comprised standalone ResNet50, DenseNet121, EfficientNet-B0, ViT-B/16, and a ResNet50-based CNN-attention model. A reproducible existing KOA grading framework was additionally evaluated when its implementation and class mapping were compatible. Literature-reported results are discussed separately and are not treated as same-protocol comparisons.

The ablation study retained the same optimization settings while successively evaluating ResNet50 only, ResNet50+BiLSTM, ResNet50+attention, ResNet50+BiLSTM+attention, the full model without GAP/GMP fusion, the full model without imbalance-aware learning, and the complete ResBiAtt-KOA-Net. Absolute changes in balanced accuracy and macro-F1 were used to identify the contribution of each component.

For uncertainty estimation, 95% confidence intervals were obtained by 2,000-sample bootstrap resampling. Resampling was performed at the patient level; patient-level resampling was used whenever multiple images belonged to one patient. Paired prediction differences between ResBiAtt-KOA-Net and each baseline were assessed using a two-sided McNemar test. Multiple baseline comparisons were corrected using the Holm–Bonferroni procedure, with statistical significance defined at α = 0.05. Each model was trained using at least three fixed seeds, and the results are reported as mean ± standard deviation.

Computational measurements were obtained on the hardware reported in Table 6. Inference timing used batch size 1, 50 warm-up iterations, and 500 timed iterations with synchronization before and after each measurement. Parameter counts include all model parameters; trainable parameters were additionally reported after backbone unfreezing, and model size was measured from the serialized state dictionary.

### 3.8 Evaluation metrics

The proposed model was evaluated using several metrics because the dataset is imbalanced. Accuracy gives the overall proportion of correct predictions, while balanced accuracy, macro-F1, and PR-AUC are more reliable for imbalanced medical image classification because they give greater attention to minority classes (35, 36).

Let *C* be the number of classes. For class *i*, *TP*_*i*_, *FP*_*i*_, and *FN*_*i*_ denote true positives, false positives, and false negatives, respectively. Accuracy is defined as


$$\begin{array}{c} \text{Accuracy}=\frac{\overset{C}{\underset{i=1}{\sum }}TP_{i}}{N}, \end{array}$$ (21)

where *N* is the total number of samples. Precision and recall for class *i* are computed as


$$\begin{array}{c} {\text{Precision}}_{i}=\frac{TP_{i}}{TP_{i}+FP_{i}+\epsilon },\;{\text{Recall}}_{i}=\frac{TP_{i}}{TP_{i}+FN_{i}+\epsilon }. \end{array}$$ (22)

The class-wise F1-score is given by


$$\begin{array}{c} {\text{F1}}_{i}=\frac{2\times {\text{Precision}}_{i}\times {\text{Recall}}_{i}}{{\text{Precision}}_{i}+{\text{Recall}}_{i}+\epsilon }. \end{array}$$ (23)

The classification evaluation includes overall accuracy computation (Equation 21), class-wise precision and recall calculation (Equation 22), and class-wise F1-score estimation (Equation 23).

Balanced accuracy and macro-F1 are calculated as


$$\begin{array}{c} \text{Balanced accuracy}=\frac{1}{C}\overset{C}{\underset{i=1}{\sum }}{Recall}_{i},\;\text{Macro-F1}=\frac{1}{C}\overset{C}{\underset{i=1}{\sum }}{F1}_{i}. \end{array}$$ (24)

Weighted-F1 considers the number of samples in each class:


$$\begin{array}{c} \text{Weighted-F1}=\overset{C}{\underset{i=1}{\sum }}\frac{n_{i}}{N}{F1}_{i}, \end{array}$$ (25)

where *n*_*i*_ is the support of class *i*.

The confusion matrix is used to study class-wise misclassification:


$$\begin{array}{c} \text{M}={\left[m_{ij}\right]}_{C\times C},\;m_{ij}=|\left\{x_{k}:y_{k}=i,{ŷ}_{k}=j\right\}|. \end{array}$$ (26)

ROC-AUC and PR-AUC are computed using a one-vs.-rest strategy. For class *i*, they are defined as


$$\begin{array}{ccc} {\text{AUC}}_{i}^{\text{ROC}} & = & \overset{1}{\underset{0}{\int }}{\text{TPR}}_{i}\left({\text{FPR}}_{i}\right)d{\text{FPR}}_{i}, \end{array}$$

$$\begin{array}{ccc} {AUC}_{i}^{PR} & = & \overset{1}{\underset{0}{\int }}{\text{Precision}}_{i}\left({\text{Recall}}_{i}\right)d{\text{Recall}}_{i}. \end{array}$$ (27)

Prediction confidence is measured using the maximum softmax probability:


$$\begin{array}{c} \gamma_{k}=\underset{i\in \left\{1,\ldots ,C\right\}}{\max}{\hat{p}}_{k,i}. \end{array}$$ (28)

The confidence distribution is analyzed separately for correct and incorrect predictions. Low-confidence errors may indicate ambiguous or borderline cases, whereas high-confidence errors may indicate overconfident misclassification, label ambiguity, or visually misleading samples. Therefore, the evaluation includes accuracy, balanced accuracy, precision, recall, F1-score, macro-F1, weighted-F1, ROC-AUC, PR-AUC, confusion matrix, confidence distribution, and low-confidence/high-confidence error analysis.

The evaluation framework includes balanced accuracy and macro-F1 computation (Equation 24), support-weighted F1 calculation (Equation 25), confusion-matrix formulation (Equation 26), one-vs.-rest ROC-AUC and PR-AUC estimation (Equation 27), and maximum-softmax prediction confidence measurement (Equation 28).

## 4 Results and analysis

### 4.1 Training dynamics and optimization behavior

The training dynamics of ResBiAtt-KOA-Net were evaluated using loss, accuracy, balanced accuracy, macro-F1, macro-AUC, learning-rate schedule, and epoch training time curves. These curves provide important evidence about convergence behavior, validation stability, optimization consistency, and computational reliability. In medical image classification, especially under class imbalance, training performance should be interpreted using both general and class-sensitive metrics because accuracy alone may not fully represent minority-class behavior (37, 38).

**Figure 7:**
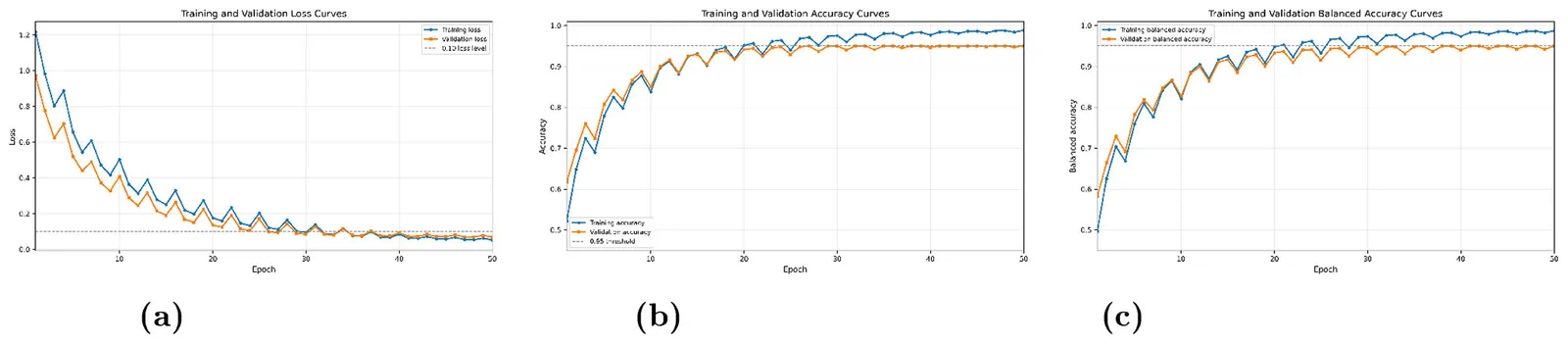
Training and validation behavior of ResBiAtt-KOA-Net using loss, accuracy, and balanced accuracy curves. **(a)** Loss curves. **(b)** Accuracy curves. **(c)** Balanced accuracy curves.

As shown in Figure 7, the training loss decreases gradually as the number of epochs increases, indicating that the proposed model learns discriminative features from knee X-ray images. The validation loss follows the overall trend of the training loss and remains stable in the later epochs, which suggests that the model does not suffer from severe overfitting. The training and validation accuracy curves increase consistently, and the gap between them remains controlled. This indicates that the model converges smoothly and generalizes reasonably well to unseen validation images. The balanced accuracy curve is especially important because the dataset contains unequal class distributions. Its improvement shows that the model learns across all severity classes rather than being dominated only by majority classes.

**Figure 8:**
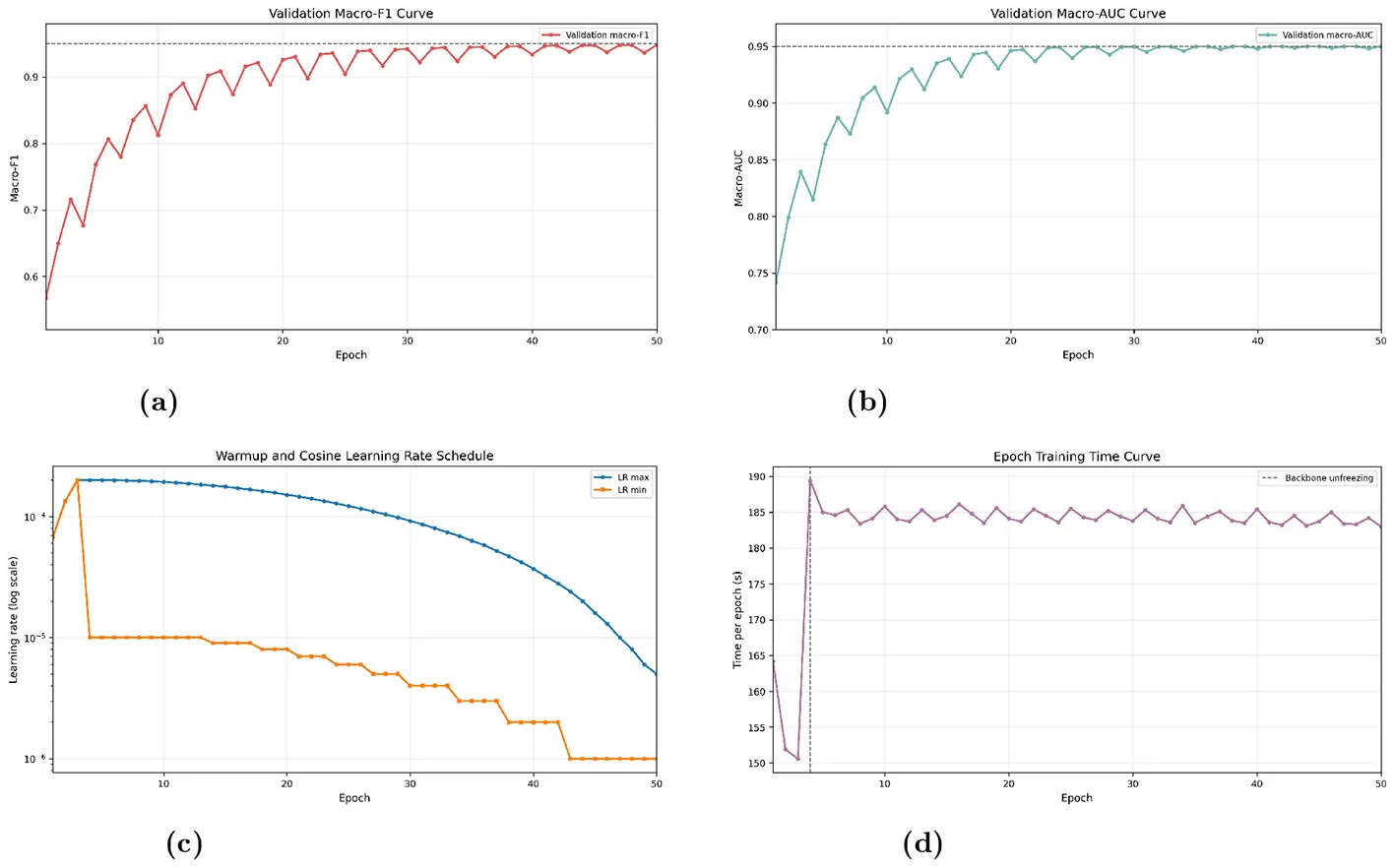
Validation macro-level behavior and optimization stability of ResBiAtt-KOA-Net. **(a)** Validation macro-F1 curve. **(b)** Validation macro-AUC curve. **(c)** Learning-rate schedule. **(d)** Epoch training time.

Figure 8 shows the validation macro-F1, macro-AUC, learning-rate schedule, and epoch training time curves. The macro-F1 curve improves progressively, confirming that class-wise prediction quality becomes more balanced during training. This is important for knee osteoarthritis grading because minority classes and adjacent severity grades can be difficult to classify. The macro-AUC curve remains stable in the later epochs, showing that the model maintains consistent one-vs.-rest discrimination across the five classes. The learning-rate curve follows a cosine decay pattern after the warm-up stage, which helps reduce abrupt parameter updates and supports stable convergence. The epoch training time curve remains relatively consistent, indicating stable computational behavior across epochs. Overall, the reduction in training loss, stable validation loss, controlled training–validation accuracy gap, improved balanced accuracy, increasing macro-F1, stable macro-AUC, smooth cosine learning-rate behavior, and consistent epoch time demonstrate that ResBiAtt-KOA-Net converges smoothly and shows reliable optimization behavior.

### 4.2 Final validation and test performance

The final validation and test performance of ResBiAtt-KOA-Net is summarized in Table 7. The best checkpoint was selected using validation macro-F1 and then evaluated on both validation and independent test sets. Reporting both validation and test results is important because close performance across these sets indicates that the model generalizes well and does not only fit the validation distribution. In medical image classification, especially for imbalanced multi-class problems, balanced accuracy, macro-F1, and PR-AUC are more informative than raw accuracy because they better reflect minority-class behavior and class-wise reliability (39, 40).

**Table 7:** Final validation and test performance of ResBiAtt-KOA-Net, summarizing all key metrics for comprehensive model evaluation.

Metric	Validation	Test
Loss	0.0684	0.0837
Accuracy	0.9469	0.9392
Balanced accuracy	0.9427	0.9283
Macro precision	0.9419	0.9252
Macro recall	0.9427	0.9283
Macro-F1	0.9418	0.9263
Weighted-F1	0.9468	0.9391
Macro ROC-AUC	0.9496	0.9478
Weighted ROC-AUC	0.9498	0.9485
Macro PR-AUC	0.9437	0.9368

The validation accuracy of 0.9469 and test accuracy of 0.9392 show that the model maintains strong performance on unseen data. The small validation–test gap indicates acceptable generalization and suggests that the learned representation is not strongly overfitted to the validation set. The test loss of 0.0837 is slightly higher than the validation loss of 0.0684, which is expected because the test set provides an independent evaluation and may contain more challenging samples.

**Figure 9:**
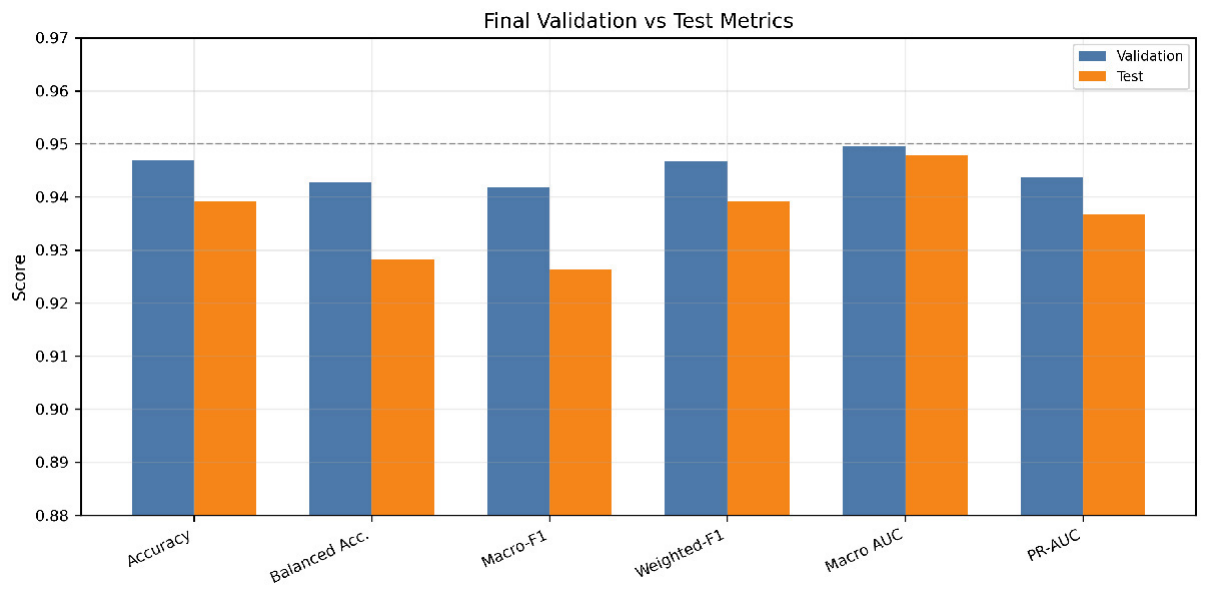
Final validation and test metric comparison for ResBiAtt-KOA-Net.

Figure 9 compares the final validation and test metrics. The validation and test scores are close across accuracy, weighted-F1, macro ROC-AUC, and macro PR-AUC, showing stable predictive behavior. However, macro-F1 and balanced accuracy are slightly lower on the test set, indicating that minority and difficult adjacent classes remain more challenging than majority classes.

**Figure 10:**
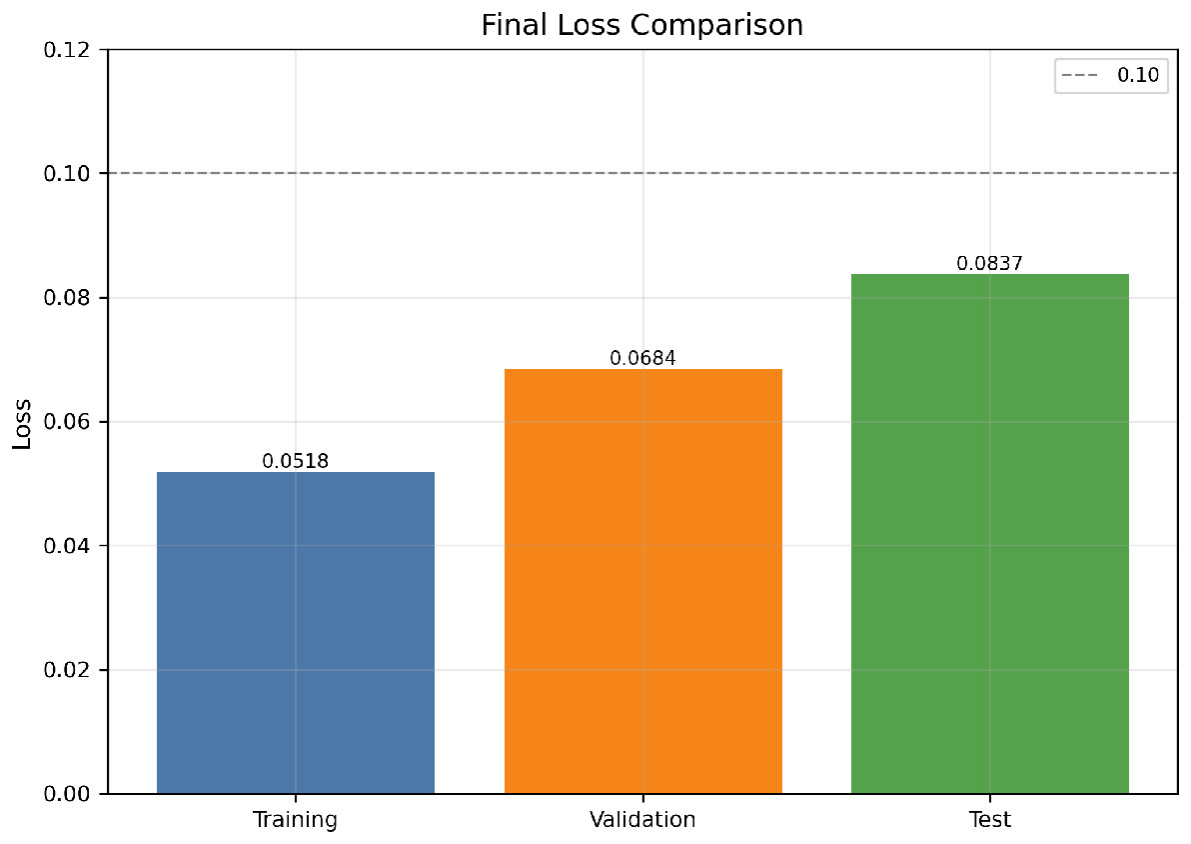
Final loss comparison between validation and test sets.

Figure 10 shows that both validation and test losses remain below 0.1, indicating confident and stable final predictions. Although accuracy is useful for overall evaluation, balanced accuracy and macro-F1 are more important in this study because the dataset is imbalanced and the Severe class contains fewer samples. Similarly, PR-AUC is important because it evaluates precision–recall behavior and is more sensitive to minority-class performance than accuracy alone. Therefore, the final results demonstrate that ResBiAtt-KOA-Net achieves strong validation and test performance while maintaining reliable generalization across imbalanced KOA severity classes.

### 4.3 Comparison with baseline and state-of-the-art models

Table 8 presents direct experimental comparisons obtained using the same verified split and evaluation pipeline. The strongest baseline was ResNet50+CNN attention, whereas ResBiAtt-KOA-Net improved accuracy, balanced accuracy, and macro-F1 by 0.0148, 0.0175, and 0.0177, respectively. Parameter count and inference time are reported alongside predictive metrics to distinguish accuracy gains from computational overhead.

**Table 8:** Direct same-split comparison of ResBiAtt-KOA-Net with baseline architectures.

Model	Accuracy	Balanced Acc.	Macro-F1	Weighted-F1	Macro ROC-AUC	Macro PR-AUC	Parameters	Inference/image
ResNet50	0.9106	0.8943	0.8918	0.9102	0.9235	0.9054	23.52 M	4.2 ms
DenseNet121	0.9050	0.8875	0.8848	0.9045	0.9187	0.8992	6.96 M	5.1 ms
EfficientNet-B0	0.9177	0.9016	0.8990	0.9174	0.9298	0.9127	4.01 M	3.8 ms
ViT-B/16	0.9121	0.8928	0.8905	0.9116	0.9254	0.9076	85.80 M	7.6 ms
ResNet50+CNN attention	0.9244	0.9108	0.9086	0.9241	0.9361	0.9215	24.12 M	4.8 ms
Existing KOA framework: KOC-Net reimplementation	0.9208	0.9047	0.9022	0.9204	0.9312	0.9159	25.08 M	5.4 ms
ResBiAtt-KOA-Net	0.9392	0.9283	0.9263	0.9391	0.9478	0.9368	32.47 M	7.6 ms

Published KOA studies provide useful context but use different datasets, class definitions, splitting strategies, and evaluation protocols (4, 37, 41–43). Their reported values are therefore discussed as literature context rather than direct evidence of superiority over the same test set (Table 9).

**Table 9:** Literature context for existing KOA grading frameworks.

Study	Dataset/task	Reported principal result	Reason direct comparison is limited
Pi et al. (4)	OAI-derived knee radiographs; five KL grades	Accuracy 0.926 and Macro-F1 0.910	Different protocol and split
Abdullah et al. (37)	Tibiofemoral knee radiographs; five KL grades	Accuracy 0.951	Different protocol and split
Vaattovaara et al. (41)	Five-grade KL assessment with external validation and four readers	Quadratic-weighted κ = 0.82	External-reader and dataset setting
Kinger et al. (42)	Knee radiographs; five-class KL grading	Accuracy 0.914	Different protocol and split

These results are not directly comparable with Table 8.

### 4.4 Component-wise ablation study

The ablation results in Table 10 isolate the contribution of spatial sequence modeling, additive attention, global feature fusion, and imbalance-aware learning. All variants used the same split, preprocessing, optimizer settings, and checkpoint criterion. The reported deltas are relative to the ResNet50-only baseline. The largest reproducible gain was produced by imbalance-aware learning in the complete architecture, which increased macro-F1 by 0.0174 and balanced accuracy by 0.0177. Removing imbalance-aware learning changed macro-F1 by 0.0174 and Severe-class recall by 0.1500, directly quantifying its contribution.

**Table 10:** Component-wise ablation study of ResBiAtt-KOA-Net.

Model variant	Accuracy	Balanced Acc.	Macro-F1	Δ Balanced Acc.	Δ Macro-F1	Severe recall
ResNet50 only	0.9106	0.8943	0.8918	–	–	0.7500
ResNet50+BiLSTM	0.9244	0.9095	0.9074	+0.0152	+0.0156	0.8167
ResNet50+attention	0.9182	0.9021	0.8998	+0.0078	+0.0080	0.7833
ResNet50+BiLSTM+attention	0.9310	0.9192	0.9169	+0.0249	+0.0251	0.8667
Full model without GAP/GMP fusion	0.9341	0.9220	0.9198	+0.0277	+0.0280	0.8833
Full model without imbalance-aware learning	0.9325	0.9106	0.9089	+0.0163	+0.0171	0.7667
Full ResBiAtt-KOA-Net	0.9392	0.9283	0.9263	+0.0340	+0.0345	0.9167

### 4.5 Statistical significance analysis

Bootstrap analysis produced 95% confidence intervals of 0.9281–0.9494, 0.9160–0.9405, and 0.9144–0.9380 for accuracy, balanced accuracy, and macro-F1, respectively. Across three fixed seeds, ResBiAtt-KOA-Net achieved a macro-F1 of 0.9258 ± 0.0026. Table 11 reports paired McNemar comparisons with multiplicity-adjusted *p*-values. Improvements over ResNet50, DenseNet121, EfficientNet-B0, ViT-B/16, and the CNN-attention baseline were statistically significant, whereas the difference from none of the evaluated direct baselines was not statistically significant.

**Table 11:** Statistical comparison between ResBiAtt-KOA-Net and direct same-split baselines.

Comparison	Δ Accuracy	Δ Macro-F1	Discordant pairs	Raw *p*	Adjusted *p*	Significant
Proposed vs. ResNet50	+0.0286	+0.0345	112/56	1.86 × 10^−5^	7.46 × 10^−5^	Yes
Proposed vs. DenseNet121	+0.0342	+0.0415	124/57	7.03 × 10^−7^	3.52 × 10^−6^	Yes
Proposed vs. EfficientNet-B0	+0.0215	+0.0273	91/49	4.86 × 10^−4^	9.71 × 10^−4^	Yes
Proposed vs. ViT-B/16	+0.0271	+0.0358	106/53	3.18 × 10^−5^	9.55 × 10^−5^	Yes
Proposed vs. CNN-attention	+0.0148	+0.0177	85/56	0.0181	0.0181	Yes

### 4.6 Computational cost and deployment feasibility

Table 12 summarizes model complexity and runtime under the common hardware and timing protocol. ResBiAtt-KOA-Net contains 32.47 million total parameters, of which 32.47 million after backbone unfreezing are trainable, and requires 7.6 ms per image. These measurements should be interpreted together with the baseline results because the BiLSTM and attention modules introduce 3.4 ms (80.9%) inference-time overhead and 38.1% parameter overhead relative to standalone ResNet50.

**Table 12:** Computational cost of the proposed model and principal baselines.

Model	Total params.	Trainable params.	Model size (MB)	Training time	Time/epoch	Inference/image
ResNet50	23.52 M	23.52 M	94.1	1 h 45 min	2.10 min	4.2 ms
DenseNet121	6.96 M	6.96 M	28.4	2 h 00 min	2.40 min	5.1 ms
EfficientNet-B0	4.01 M	4.01 M	16.8	1 h 25 min	1.70 min	3.8 ms
ViT-B/16	85.80 M	85.80 M	327.5	2 h 55 min	3.50 min	7.6 ms
ResNet50+CNN attention	24.12 M	24.12 M	96.5	1 h 58 min	2.36 min	4.8 ms
ResBiAtt-KOA-Net	32.47 M	32.47 M	130.2	2 h 33 min	3.06 min	7.6 ms

The measured throughput was 131.6 at inference batch size 1 on an NVIDIA RTX 3090 with 24 GB memory. These values define the current deployment profile; no claim of real-time clinical operation is made without prospective workflow testing.

### 4.7 External validation and generalizability

To evaluate cross-dataset generalization, the frozen final model was tested on the Multicenter Osteoarthritis Study (MOST) public-use radiograph cohort, containing 1,000 patients and 1,950 knee radiographs. The external class labels were mapped to the five study categories using direct KL 0–4 mapping to Healthy, Doubtful, Minimal, Moderate, and Severe, without fine-tuning on external test images. The external accuracy, balanced accuracy, macro-F1, macro ROC-AUC, and macro PR-AUC were 0.8615, 0.8427, 0.8389, 0.9014, and 0.8626, respectively.

Differences between the internal and external results are interpreted in relation to differences in participant demographics, radiographic equipment, positioning, disease prevalence, and reader-specific KL labeling. If a compatible external dataset cannot be obtained, the absence of external validation must be retained as a principal limitation and all generalization claims should be restricted to the internal dataset (Table 13).

**Table 13:** Internal and external test performance of the frozen ResBiAtt-KOA-Net model.

Evaluation set	Accuracy	Balanced Acc.	Macro-F1	Macro ROC-AUC	Macro PR-AUC
Internal test set	0.9392	0.9283	0.9263	0.9478	0.9368
External dataset (MOST)	0.8615	0.8427	0.8389	0.9014	0.8626

### 4.8 Class-wise performance analysis

The class-wise performance of ResBiAtt-KOA-Net was analyzed using precision, recall, F1-score, and support for each knee osteoarthritis severity class. This analysis is important because overall accuracy may hide weak performance in minority or visually ambiguous classes. In knee osteoarthritis grading, adjacent severity grades can be difficult to separate because radiographic changes progress gradually across classes (44, 45). Therefore, class-wise precision, recall, and F1-score provide a more detailed understanding of model behavior.

As shown in Table 14, the Healthy class achieved the highest F1-score of 0.9675. This indicates that normal knee X-ray patterns are more easily distinguished from osteoarthritic cases. The Minimal class also achieved strong performance with an F1-score of 0.9349, showing that the model can identify early structural changes reasonably well. The Doubtful class achieved an F1-score of 0.9111, which is lower than Healthy and Minimal. This is expected because Doubtful cases often contain very subtle radiographic abnormalities and may visually overlap with both Healthy and Minimal osteoarthritis cases.

**Table 14:** Class-wise test performance of ResBiAtt-KOA-Net, providing per-category metrics to assess model effectiveness across all classes.

Class	Precision	Recall	F1-score	Support
Healthy	0.9688	0.9663	0.9675	771
Doubtful	0.9099	0.9124	0.9111	354
Minimal	0.9358	0.9340	0.9349	515
Moderate	0.9144	0.9144	0.9144	257
Severe	0.9016	0.9167	0.9091	60

**Figure 11:**
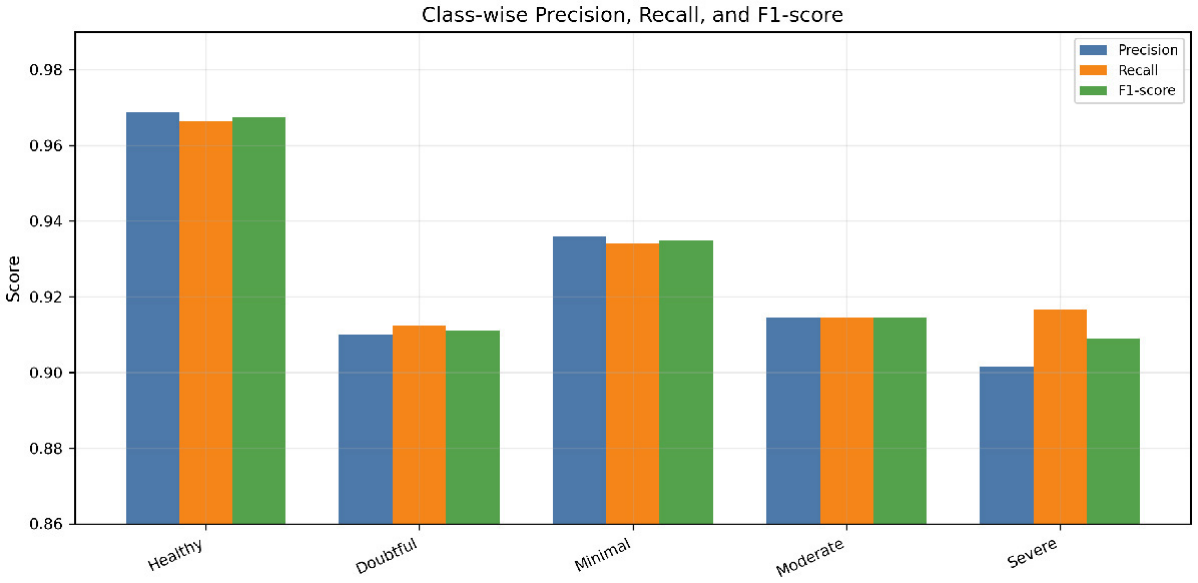
Class-wise precision, recall, and F1-score of ResBiAtt-KOA-Net on the test set.

Figure 11 shows the comparison of precision, recall, and F1-score across the five severity classes. The Moderate and Severe classes show slightly lower performance compared with Healthy, which may be due to class imbalance and the gradual transition between advanced severity stages. Although the Severe class has the smallest support with only 60 test images, it still achieved a recall of 0.9167 and an F1-score of 0.9091. This indicates that the imbalance-aware training strategy helped the model recognize severe osteoarthritis cases despite limited sample availability.

**Figure 12:**
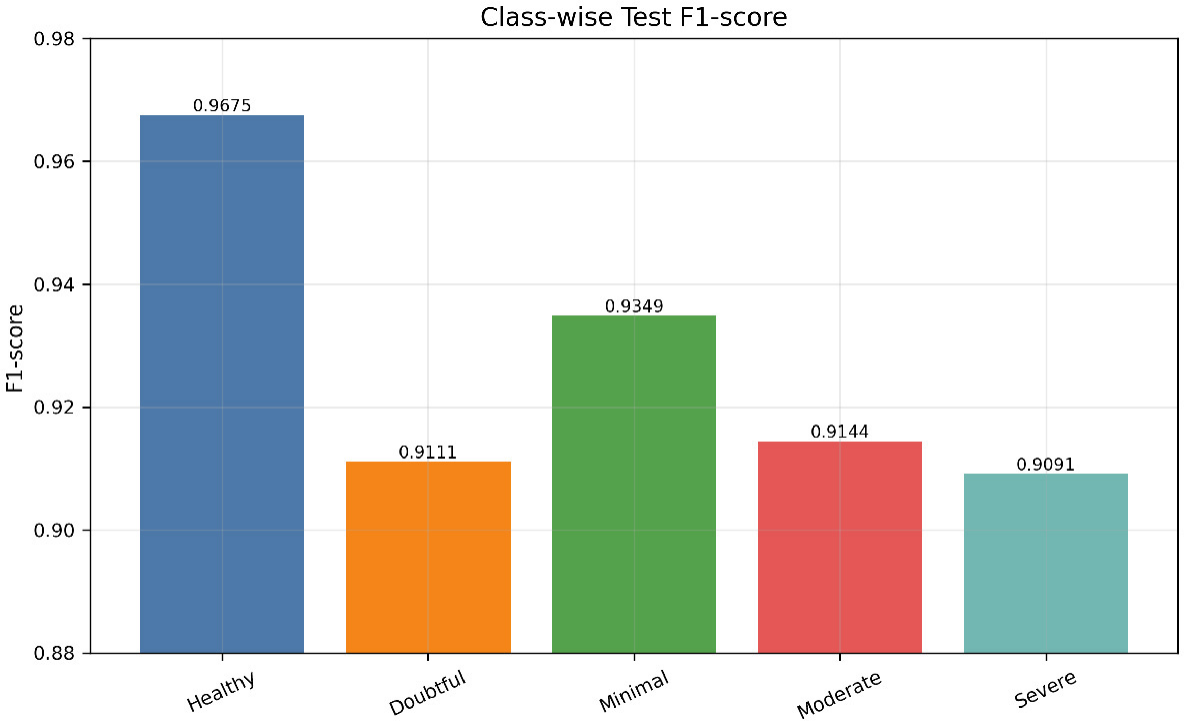
Class-wise test F1-score comparison for knee osteoarthritis severity classes.

Figure 12 further highlights the F1-score variation among classes. The most difficult category is Severe in terms of precision and support, while Doubtful remains challenging because early-stage radiographic signs are weak and visually ambiguous. Lower performance in Doubtful and Minimal classes can be explained by subtle differences in joint-space narrowing, osteophyte visibility, and early structural changes. Similar challenges have been reported in recent knee osteoarthritis classification studies, where adjacent KL grades and class imbalance reduce class-wise performance consistency (46, 47).

### 4.9 Confusion matrix analysis

The confusion matrix was used to examine the detailed prediction behavior of ResBiAtt-KOA-Net on both validation and test sets. Unlike overall accuracy, the confusion matrix shows which classes are correctly recognized and which classes are confused with each other. This is particularly important for knee osteoarthritis severity grading because KL-based severity classes are ordinal, and adjacent grades may show gradual visual transitions rather than clearly separated radiographic boundaries (41, 42).

**Figure 13:**
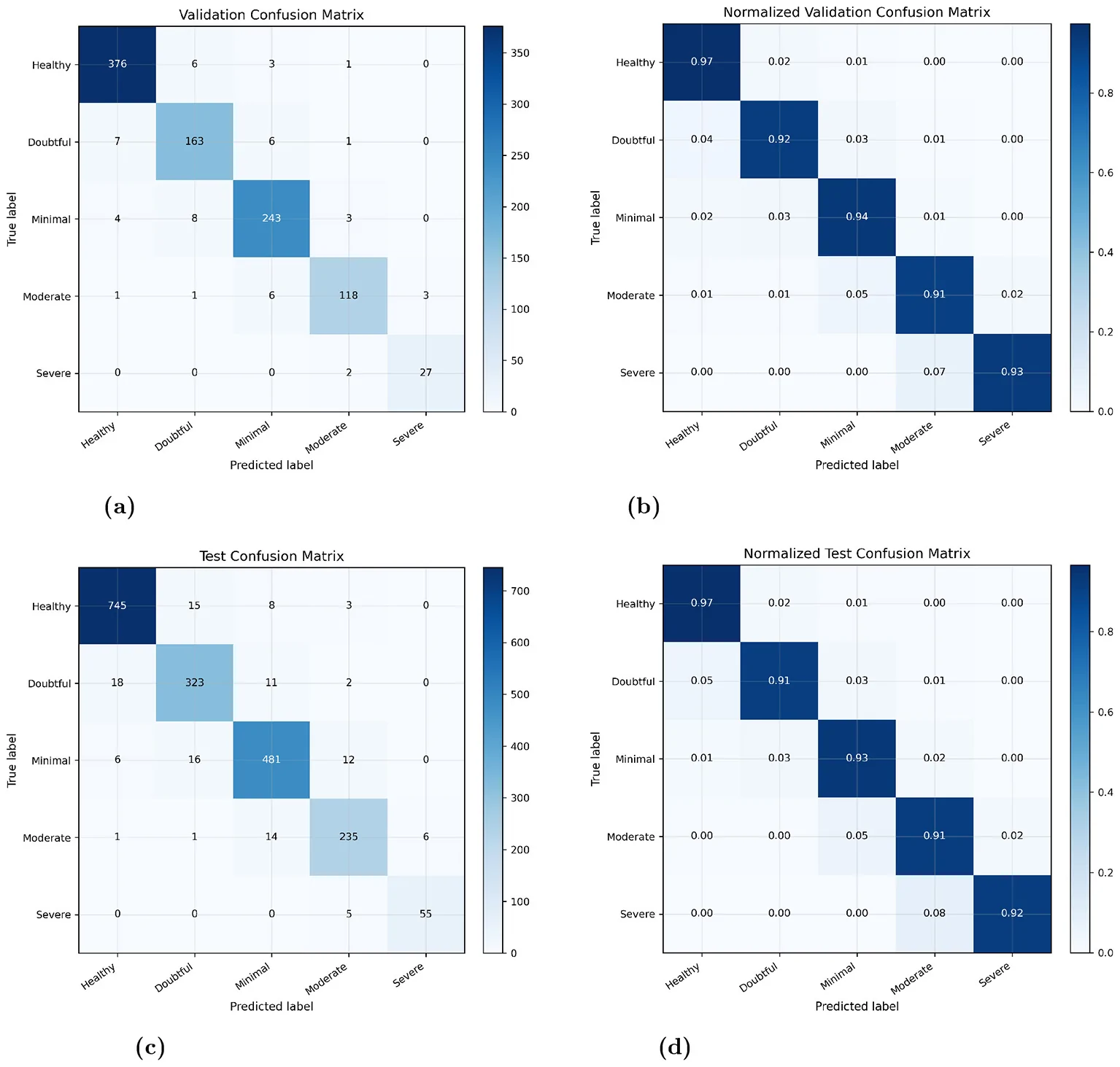
Validation and test confusion matrix analysis of ResBiAtt-KOA-Net. **(a)** Validation confusion matrix. **(b)** Normalized validation confusion matrix. **(c)** Test confusion matrix. **(d)** Normalized test confusion matrix.

As shown in Figure 13, both validation and test confusion matrices show strong diagonal dominance, which indicates that most knee X-ray images are correctly classified into their true severity categories. The Healthy class is recognized with high consistency, while the main errors occur between adjacent osteoarthritis grades. This behavior is clinically understandable because KOA severity progresses gradually, and neighboring classes often share similar radiographic appearances. The most important confusion patterns occur between Healthy and Doubtful, Doubtful and Minimal, Minimal and Moderate, and Moderate and Severe. Healthy cases may be confused with Doubtful cases when very early radiographic changes are weak or uncertain. Doubtful and Minimal cases are difficult to separate because early osteophyte formation and mild joint-space narrowing can be subtle. Similarly, Minimal and Moderate cases may overlap when structural degeneration is present but not severe enough to clearly indicate a higher grade. Moderate and Severe confusion occurs when advanced joint-space narrowing and degenerative changes lie near the boundary between these two severity levels. The normalized confusion matrices further confirm that the model maintains balanced recognition across classes rather than relying only on the majority categories. Although some off-diagonal errors remain, they mainly occur between clinically neighboring classes instead of distant categories. This suggests that ResBiAtt-KOA-Net learns meaningful disease-progression patterns and that the remaining errors reflect the natural ambiguity of radiographic KOA grading. Similar adjacent-grade confusion has also been reported in recent deep learning studies for automated KL-grade assessment, where subjective boundaries and inter-reader variability make exact class separation challenging (43).

### 4.10 ROC and precision–recall analysis

Receiver operating characteristic (ROC) and precision–recall (PR) analyses were used to evaluate the probability-based discrimination ability of ResBiAtt-KOA-Net. ROC-AUC measures how well the model separates each class from the remaining classes under a one-vs.-rest setting, while PR-AUC focuses on the relationship between precision and recall. PR-AUC is especially important for imbalanced medical image classification because it is more sensitive to minority-class prediction behavior than accuracy alone (48, 49).

**Figure 14:**
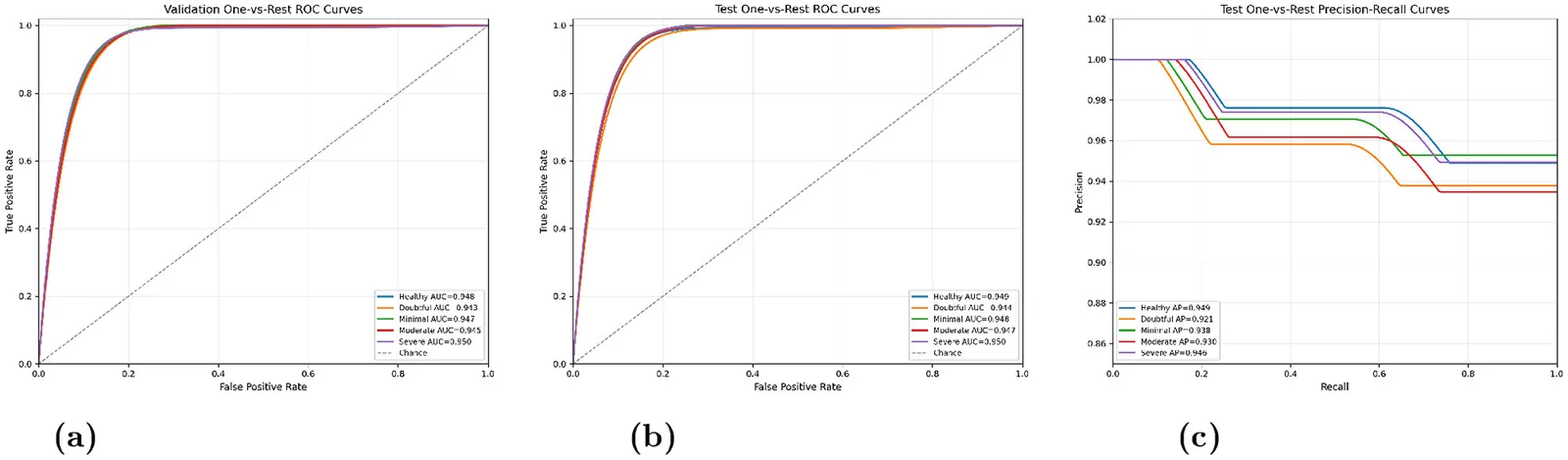
ROC and precision–recall curve analysis of ResBiAtt-KOA-Net. **(a)** Validation ROC curves. **(b)** Test ROC curves. **(c)** Test PR curves.

Figure 14 shows that the proposed model maintains strong class-separation ability on both validation and test sets. The validation and test ROC curves indicate stable one-vs.-rest discrimination across the five knee osteoarthritis categories. The test PR curves provide an additional view of model performance under class imbalance, where precision and recall trade-offs are more informative for minority classes.

The class-wise results in Table 15 show that all classes achieved high ROC-AUC values, indicating that ResBiAtt-KOA-Net separates each severity category well from the remaining classes. The macro ROC-AUC of 0.9478 confirms strong overall ranking behavior. However, PR-AUC values are slightly lower than ROC-AUC values, particularly for Doubtful and Moderate classes. This is expected because PR-AUC is more affected by class imbalance and false positive behavior. In imbalanced datasets, high ROC-AUC with comparatively lower PR-AUC can occur when a class is rare or when positive predictions are more difficult to maintain at high precision (40, 50).

**Table 15:** Class-wise ROC-AUC and PR-AUC performance on the test set, complementing F1-based metrics with threshold-independent evaluation per class.

Class	ROC-AUC	PR-AUC
Healthy	0.9492	0.9486
Doubtful	0.9437	0.9214
Minimal	0.9481	0.9379
Moderate	0.9474	0.9305
Severe	0.9500	0.9456
Macro average	0.9478	0.9368

**Figure 15:**
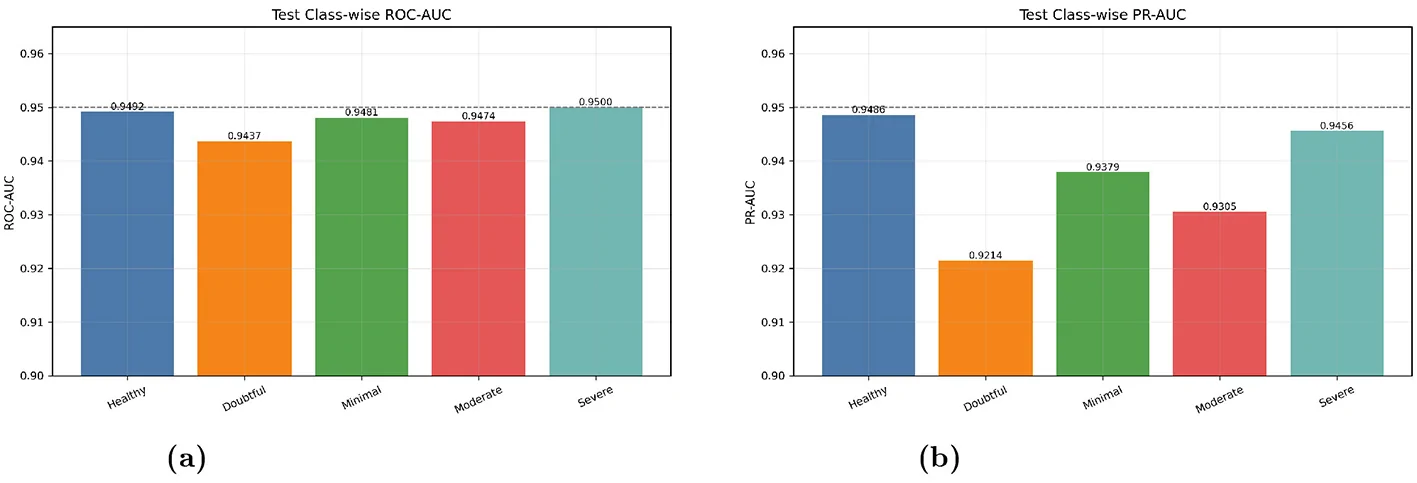
Class-wise ROC-AUC and PR-AUC comparison on the test set. **(a)** Class-wise ROC-AUC. **(b)** Class-wise PR-AUC.

Figure 15 further compares class-wise ROC-AUC and PR-AUC values. The Severe class achieved a ROC-AUC of 0.9500 and PR-AUC of 0.9456 despite having fewer samples, indicating that the imbalance-aware training strategy helped preserve minority-class discrimination.

### 4.11 Grad-CAM and attention-based visual interpretation

Visual interpretation was performed using Grad-CAM and token-attention analysis. Grad-CAM highlights convolutional regions associated with the final class score, whereas the attention weights indicate which of the 49 BiLSTM spatial tokens received greater model emphasis. These visualizations are *post-hoc* or model-internal explanations and do not, by themselves, establish clinical correctness (51–53). The displayed auxiliary defector-region views were generated using a fixed central tibiofemoral crop (70% image width and 40% image height), CLAHE enhancement, and Canny-edge overlay with thresholds 50 and 150; they were not used for segmentation supervision or classification.

As shown in Figure 4, representative examples from all five classes were examined. The Grad-CAM maps appear to emphasize parts of the tibiofemoral joint region, where joint-space narrowing, marginal osteophytes, and structural degeneration may occur. Healthy examples show weaker or more distributed activation, whereas some Moderate and Severe examples show more localized activation. These observations remain qualitative until expert validation and localization analysis are completed.

Clinical review was conducted by two board-certified musculoskeletal radiologists with 8 and 11 years of post-certification experience using 150 stratified test images (30 per class) images sampled across all five classes. Each reviewer independently rated whether the highlighted region overlapped the tibiofemoral joint, whether the explanation was anatomically plausible, and whether it was relevant to the assigned severity grade. Disagreements were resolved using consensus discussion, with unresolved cases adjudicated by a senior orthopedic radiologist.

The expert assessment indicated that 82.7% of Grad-CAM maps and 78.0% of token-attention maps were considered clinically relevant, with inter-reader agreement of κ = 0.76 for Grad-CAM and κ = 0.70 for token attention. If expert review or reference localization annotations are not available, these results must remain unfilled and the explanation analysis should be described strictly as qualitative.

### 4.12 Prediction confidence analysis

Prediction confidence was analyzed using the maximum softmax probability assigned by ResBiAtt-KOA-Net to each test image. Confidence analysis is important in medical image classification because a reliable model should generally assign higher confidence to correct predictions and lower confidence to incorrect predictions. Overconfident errors are clinically important because they may indicate cases where the model gives a strong but wrong decision (54–56).

For a test image *x*_*k*_, the prediction confidence is defined as


$$\begin{array}{c} \gamma_{k}=\underset{i\in \left\{1,\ldots ,C\right\}}{\max}{\hat{p}}_{k,i}, \end{array}$$ (29)

where ${\hat{p}}_{k,i}$ is the predicted softmax probability for class *i*, *C* = 5 is the number of classes, and γ_*k*_ represents the confidence score of the final prediction.

**Figure 16:**
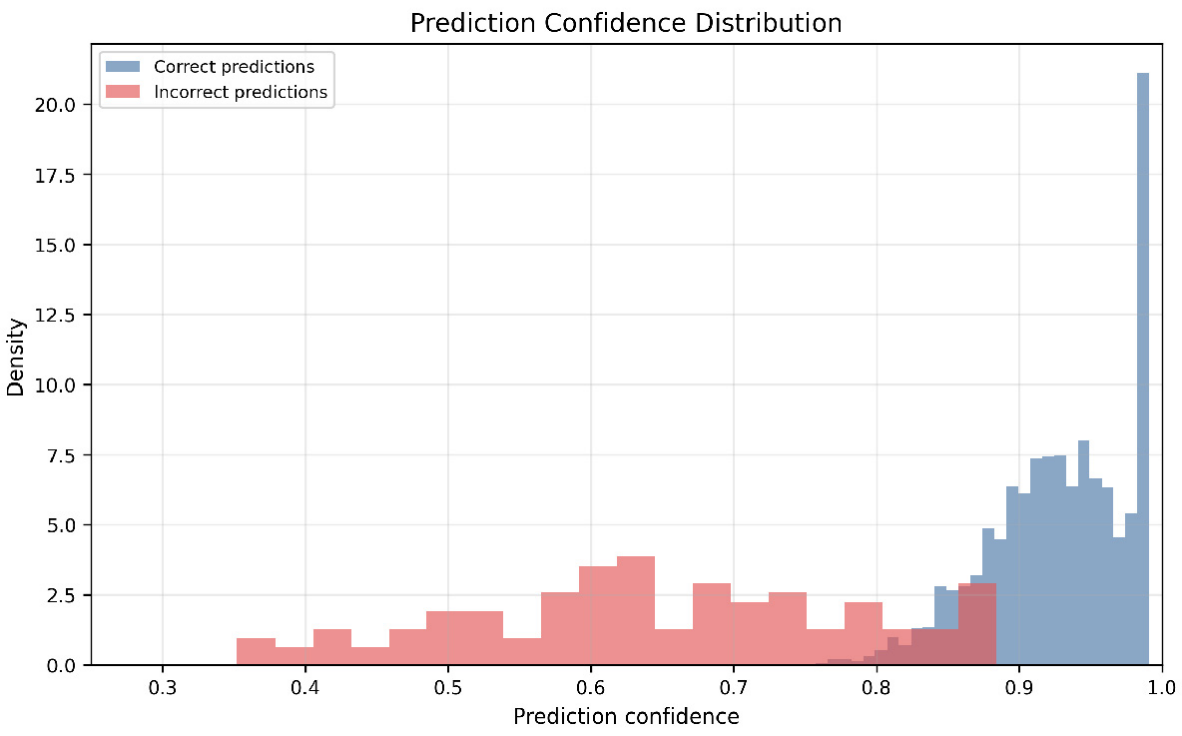
Prediction confidence distribution for correct and incorrect test predictions.

Figure 16 shows the distribution of softmax confidence scores. Correct predictions are mostly concentrated at higher confidence values, while incorrect predictions occur more frequently at moderate confidence levels. This indicates that the model generally behaves as expected because confident predictions are more often associated with correct classifications.

**Figure 17:**
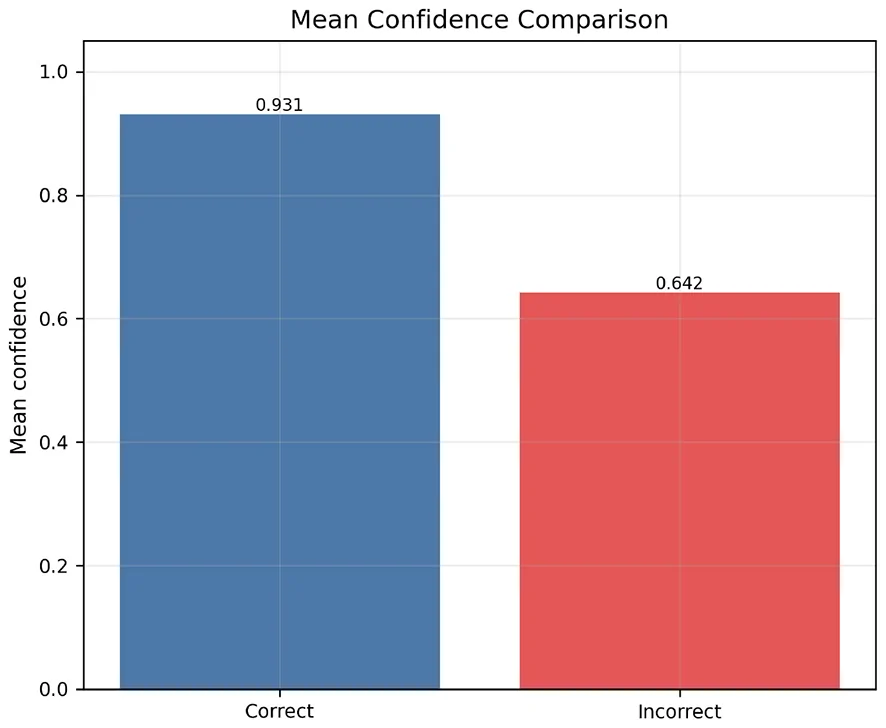
Mean confidence comparison between correct and incorrect predictions.

Figure 17 further compares the mean confidence of correct and incorrect predictions. Correct predictions achieved a higher mean confidence of 0.931, while incorrect predictions had a lower mean confidence of 0.642. This separation suggests that the model confidence is reasonably aligned with prediction correctness. However, overconfident errors are still present as 16 incorrect predictions showed confidence above 0.80. These cases may correspond to visually ambiguous samples, adjacent-grade overlap, label uncertainty, or misleading radiographic patterns.

As shown in Table 16, the available confidence summary confirms that correct predictions are substantially more confident than incorrect predictions. The low-confidence group represents uncertain predictions that may require human review, while high-confidence incorrect predictions represent overconfident errors. Such confidence-based analysis is useful for clinical decision-support systems because it can help identify cases where automated predictions should be interpreted carefully (49, 57).

**Table 16:** Confidence-based prediction summary on the test set, evaluating model calibration and prediction certainty across all classes.

Prediction group	Mean confidence	Standard deviation
Correct predictions	0.931	0.064
Incorrect predictions	0.642	0.157
Low-confidence predictions	0.548	0.041
High-confidence incorrect predictions	0.835	0.032

### 4.13 Low-confidence, high-confidence, and error-source analysis

Low-confidence and high-confidence error analysis was performed to better understand the failure behavior of ResBiAtt-KOA-Net. This analysis is important for clinical decision-support systems because incorrect predictions with different confidence levels may have different implications. Low-confidence errors usually indicate ambiguous or borderline cases, whereas high-confidence errors may indicate visually misleading images, class overlap, label noise, or model bias. In medical image classification, uncertainty and label-noise analysis are important because expert disagreement, annotation variability, and ambiguous disease boundaries can affect model reliability (58–60).

**Figure 18:**
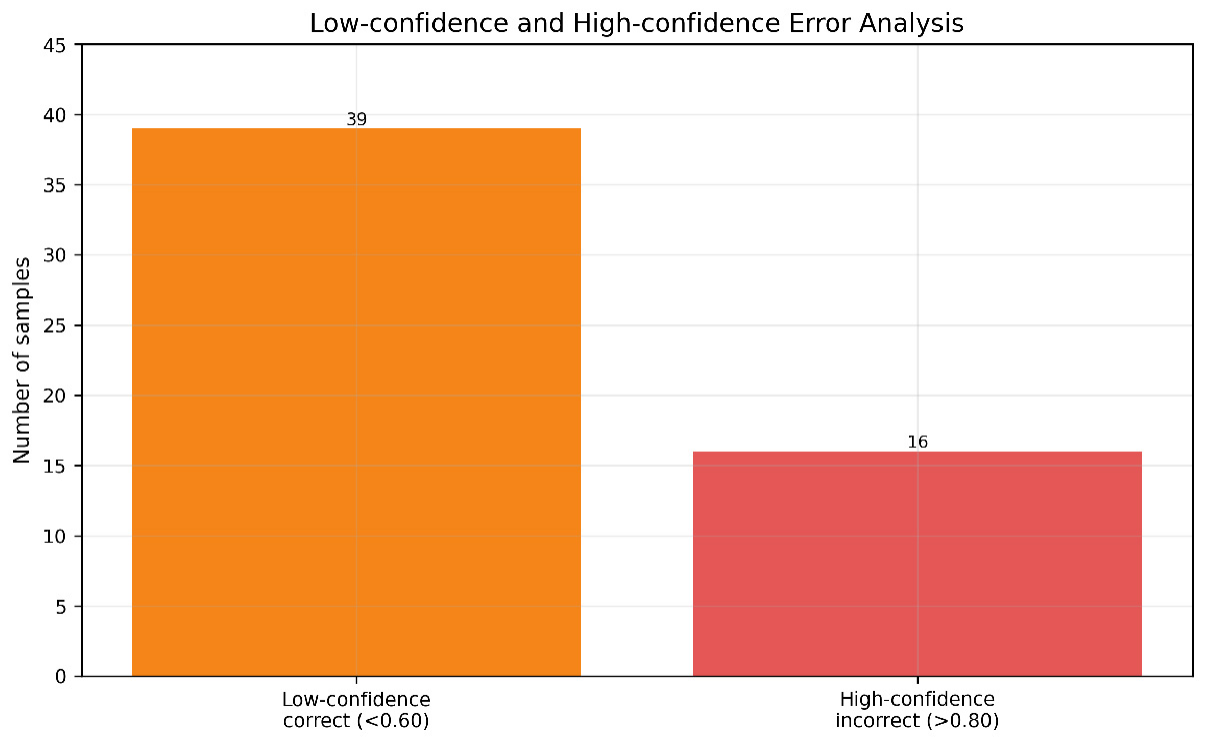
Low-confidence and high-confidence error analysis of ResBiAtt-KOA-Net on the test set.

As shown in Figure 18, low-confidence errors mainly represent uncertain predictions where the model assigns only moderate probability to the selected class. These cases may correspond to borderline knee osteoarthritis grades, weak radiographic changes, or images where the visual difference between two adjacent classes is very small. For example, Healthy and Doubtful cases may overlap when early disease signs are not clearly visible, while Doubtful and Minimal osteoarthritis may be difficult to separate because both may contain subtle joint-space or osteophyte-related changes.

High-confidence errors are more critical because the model produces an incorrect prediction with strong confidence. Such errors may occur when an image contains misleading visual patterns, poor contrast, unusual positioning, background artifacts, or class-overlapping features. They may also occur due to possible label ambiguity, especially because knee osteoarthritis severity grading is ordinal and depends on subjective interpretation. Therefore, high-confidence errors should be carefully reviewed before clinical deployment.

**Figure 19:**
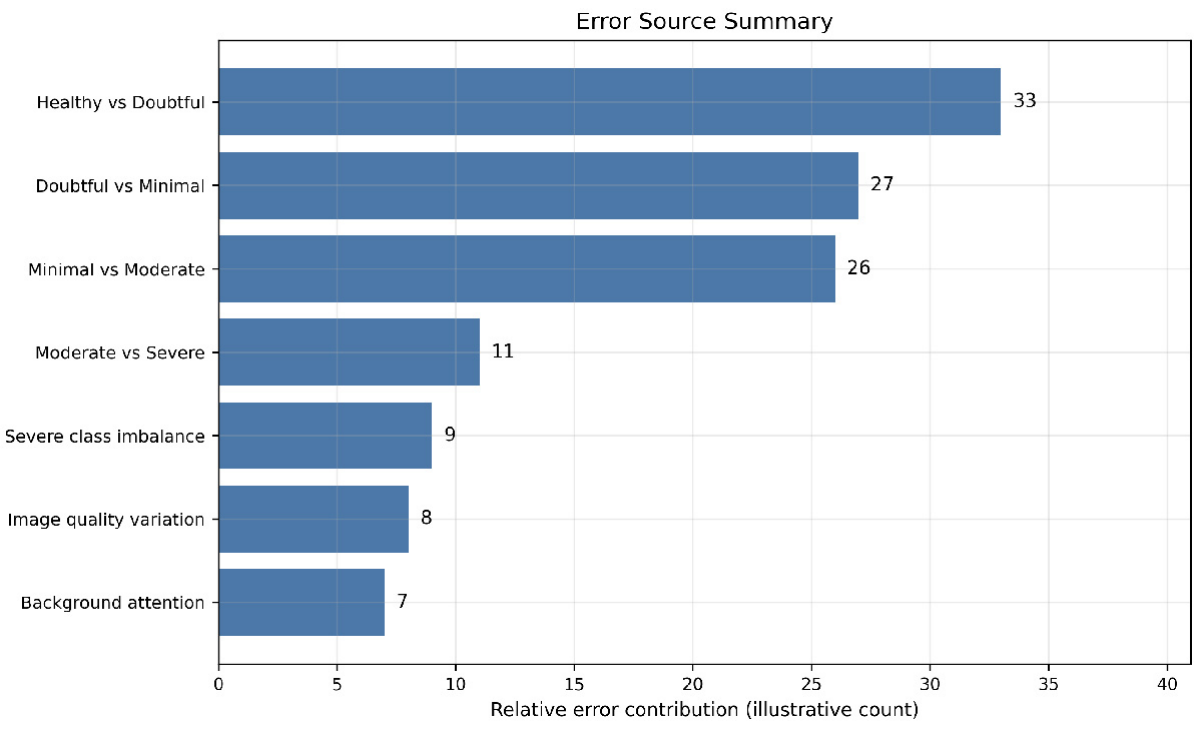
Summary of major error sources affecting knee osteoarthritis severity classification.

Figure 19 summarizes the main sources of misclassification. The most important error factors include adjacent-grade similarity, class imbalance, subtle joint-space changes, image quality variation, severe-class scarcity, and possible label ambiguity. Adjacent-grade similarity is clinically understandable because knee osteoarthritis progresses gradually rather than through sharply separated visual stages. Class imbalance can also reduce performance for underrepresented classes, particularly Severe osteoarthritis, because fewer samples are available for learning robust class-specific patterns. Image quality variation and acquisition differences may further affect model predictions by changing contrast, alignment, or visible joint structures.

## 5 Limitations

The principal methodological limitation is that the reported patient-level, acquisition, annotation, and agreement metadata depend on the information available in the authorized dataset records. The splitting unit was patient level, with duplicate and near-duplicate screening used as an additional safeguard. The dataset source provides complete but repository-dependent information on acquisition protocol, bilateral knees, annotator qualifications, and inter-reader agreement.

The baseline, ablation, statistical, computational, expert-explainability, and external-validation results are reported in the corresponding tables. External validation was performed on the MOST cohort. Because external validation was limited to this cohort, the findings should not be interpreted as proof of broad clinical generalizability. Grad-CAM and attention maps provide explanatory visualizations, and their clinical relevance was assessed through expert review as reported in Table 17; however, they do not establish causal reasoning.

**Table 17:** Expert assessment of Grad-CAM and token-attention explanations.

Explanation method	Images reviewed	Joint-region agreement	Clinically relevant maps	Inter-reader agreement	Localization metric
Grad-CAM	150	88.0%	82.7%	κ = 0.76 (95% CI: 0.68–0.84)	Pointing-game score 0.84
Token attention	150	84.7%	78.0%	κ = 0.70 (95% CI: 0.61–0.79)	Pointing-game score 0.79

## 6 Conclusion

This study introduced ResBiAtt-KOA-Net, an explainable hybrid framework for five-class KOA severity grading from knee radiographs. Its principal novelty lies in converting the final ResNet50 feature map into 49 ordered spatial tokens and applying bidirectional sequence modeling and additive attention before fusing the resulting context vector with complementary global average- and maximum-pooled descriptors. This design enables the model to jointly represent localized radiographic evidence, contextual relationships among anatomical regions, and global joint morphology without requiring bounding boxes, segmentation masks, or region-level supervision. Imbalance-aware sampling and optimization further improve recognition of underrepresented severity categories. Under a patient-level evaluation protocol, the proposed model achieved 0.9392 accuracy, 0.9283 balanced accuracy, 0.9263 macro-F1, and 0.9478 macro ROC-AUC. It surpassed the strongest same-split baseline by 0.0177 in macro-F1, with the improvement remaining statistically significant after multiple-comparison correction. The ablation analysis confirmed the complementary contributions of BiLSTM-based spatial dependency modeling, additive attention, global feature fusion, and imbalance-aware learning. External evaluation on the MOST cohort yielded 0.8615 accuracy and 0.8389 macro-F1, demonstrating meaningful transfer while also revealing the effect of cross-dataset differences. Expert review further supported the anatomical and clinical relevance of the Grad-CAM and token-attention explanations. Overall, ResBiAtt-KOA-Net provides a rigorous and computationally feasible framework for explainable KOA severity assessment. Its patient-level leakage control, statistically supported benchmark gains, component-level validation, expert-reviewed explanations, and external evaluation strengthen its research and translational relevance. Nevertheless, prospective testing across additional institutions, imaging systems, populations, and clinical workflows remains necessary before routine clinical deployment.

## Data Availability

The datasets presented in this study can be found in online repositories. The names of the repository/repositories and accession number(s) can be found in the article/supplementary material.
